# Isolation and characterization of a lytic bacteriophage against *Pseudomonas aeruginosa*

**DOI:** 10.1038/s41598-021-98457-z

**Published:** 2021-09-29

**Authors:** Sonika Sharma, Sibnarayan Datta, Soumya Chatterjee, Moumita Dutta, Jhuma Samanta, Mohan G. Vairale, Rajeev Gupta, Vijay Veer, Sanjai K. Dwivedi

**Affiliations:** 1 Defence Research Laboratory (DRL-DRDO), Tezpur, Assam India; 2 National Institute of Cholera and Enteric Diseases (ICMR-NICED), Kolkata, West Bengal India

**Keywords:** Microbiology, Bacteriophages, Biotechnology, Environmental biotechnology, Sequencing

## Abstract

In recent years, the use of bacteriophages (or 'phages') against multidrug-resistant (MDR) bacteria including *Pseudomonas aeruginosa* has drawn considerable attention, globally. In this work, we report the isolation and detailed characterization of a highly lytic Pseudomonasphage DRL-P1 isolated from wastewater. Under TEM, DRL-P1 appeared as a member of the phage family *Myoviridae*. DRL-P1 featured rapid adsorption (~ 5 min), short-latency (~ 30 min), and large burst size (~ 100 PFU per infected cell). DRL-P1 can withstand a wide temperature range (4 °C to 40 °C) and pH (5.0 to 10.0) conditions. The 66,243 bp DRL-P1 genome (MN564818) encodes at least 93 ORFs, of which 36 were functionally annotated based on homology with similar phage proteins available in the databases. Comparative analyses of related genomes suggest an independent evolutionary history and discrete taxonomic position of DRL-P1 within genus *Pbunavirus*. No toxin or antibiotic resistance genes was identified. DRL-P1 is tolerant to lyophilization and encapsulation techniques and retained lytic activity even after 18 months of storage. We also demonstrated decontaminating potentials of DRL-P1 in vitro, on an artificially contaminated cover-slip model. To the best of our knowledge, this is the first Pbunavirus to be reported from India. Our study suggests DRL-P1 as a potential candidate for various applications.

## Introduction

For several decades, antibiotics have played an important role as therapeutic and prophylactic in healthcare clinics, veterinary, agriculture, food processing industries, etc. However, indiscriminate use of antibiotics has led to the evolution of various escape mechanisms in the bacteria, rendering them resistant to most of the available antibiotics^1,2^. Additionally, the decline in research and development of new antibiotics has left us with a very limited choice of weapons against pathogenic bacteria^1^. It is predicted that, by 2050, antibiotic resistance will result in 10 million deaths, each year^3^. Patients with chronic illnesses are more likely to develop antibiotic-resistant infections, therefore; the treatment risk associated with immune-compromised individuals is much higher than the normal patients.

In recent years, increasing morbidity and mortality due to infections with multidrug-resistant ESKAPE pathogens (*Enterococcus faecium, Staphylococcus aureus, Klebsiella pneumoniae, Acinetobacter baumannii, Pseudomonas aeruginosa,* and *Enterobacter* species) have become a serious concern, globally^4^. Among the ESKAPE pathogens, carbapenem-resistant *A*. *baumannii*, *P. aeruginosa*, and *Enterobacteriaceae* have been classified by the WHO as ‘*Priority 1 critical pathogens*’ for which R&D in new antibacterials is urgently required^5^. Because of the rapidly increasing cases of antimicrobial resistance, India too has recently declared *K. pneumoniae, E. coli, A. baumannii,* and *P. aeruginosa* as the ‘*critical priority*’ pathogens^6^. Nevertheless, the recent success of phage therapy in cases of difficult-to-treat ESKAPE infection, through administration of lytic bacteriophages has proved beyond doubt, the enormous potentials of bacteriophages in the war against this pathogens^7–13^.

Among the ESKAPE pathogens, *P. aeruginosa* is a gram-negative opportunistic pathogen, predominantly found in hospitals, animal farms, slaughterhouses, soil, aquatic environment, and sewage water. *P. aeruginosa* is notorious for being the major cause of death by nosocomial infections, especially in patients with severe wounds, causing sepsis in immunosuppressed patients, chronic lung infections in patients with cystic fibrosis, and chronic obstructive lung disease, bladder-catheter associated chronic infections in the urinary tract and ventilator-associated serious pneumonia^14,15^. In most cases, treatment of *P. aeruginosa* is very challenging owing to its multiple mechanisms to resist antibiotics and ability to form antibiotic-resistant biofilms^14,15^. The presence of flagella and type IV pili in *P. aeruginosa* allows motility on solid or semi-solid surfaces, resulting in cross-contamination of surfaces and tools, especially in clinical settings^16,17^.

In the scenario of rising antibiotic resistance among serious pathogens, the re-emergence of phage therapy has brought hope and a paradigm shift in the development of a new class of antibacterial. Harnessing the lytic activity of phages against specific bacteria has shown to be an effective approach, albeit with certain limitations. Phages are considered safer to humans and environment-friendly, as compared to the conventional chemical-based antibacterial agents. Although bacteriophages are ubiquitous in our ecosystem, sewage water receiving human fecal matter is considered as an excellent reservoir of phages against various pathogenic bacteria, prevalent in a given population^18^. Recently, researchers have demonstrated the potential application of phages isolated from sewage and other sources in the treatment of certain superbug infections including *P. aeruginosa* infection, that were otherwise untreatable using conventional antibiotics^7–10^.

In comparison to the world literature, only a few studies are available on Pseudomonasphages isolated from India^19–25^. Despite demonstrating the lytic capabilities of the isolated phages, most of these studies lacked essential characterization of the phage, which is critical for ascertaining the suitability of the phages for therapeutic and other applications^26^. In this manuscript, we describe the results of phenotypic and genotypic characterization of the novel *P. aeruginosa* phage DRL-P1 that we isolated from a wastewater source in northeastern India and demonstrate its lytic efficiency against MDR *P. aeruginosa*.

## Results

### Plaque and phage morphology of DRL-P1

**Figure 1:**
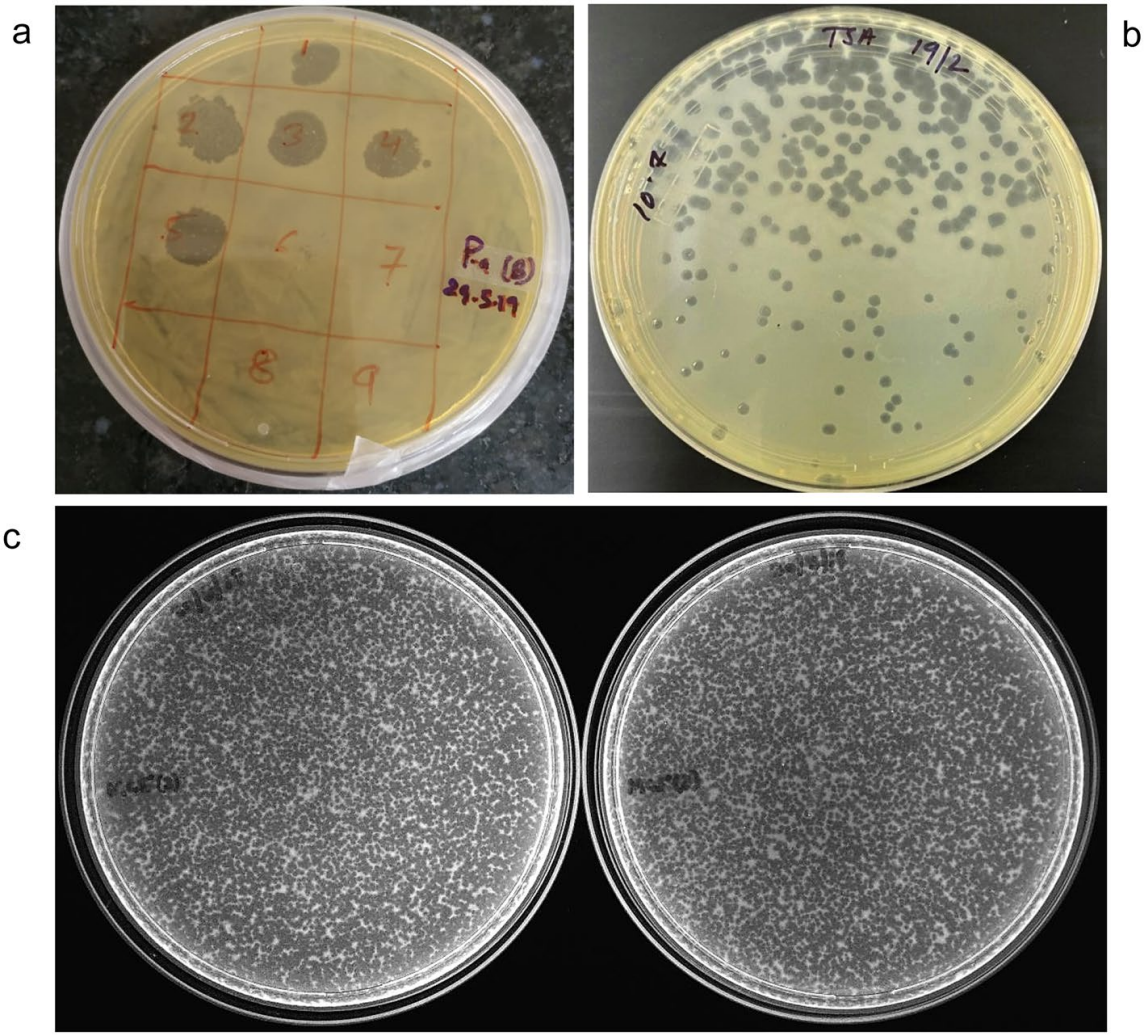
Plaques of Pseudomonasphage DRL-P1 on *P. aeruginosa* lawn*.* (**a**) A clear zone of lysis was observed in spot-test. (**b**) Clear plaques were observed after double layer agar plating of enriched phages (**c**) ‘*Web-pattern*’ plates for preparation of high titer phage lysate for different studies.

**Figure 2:**
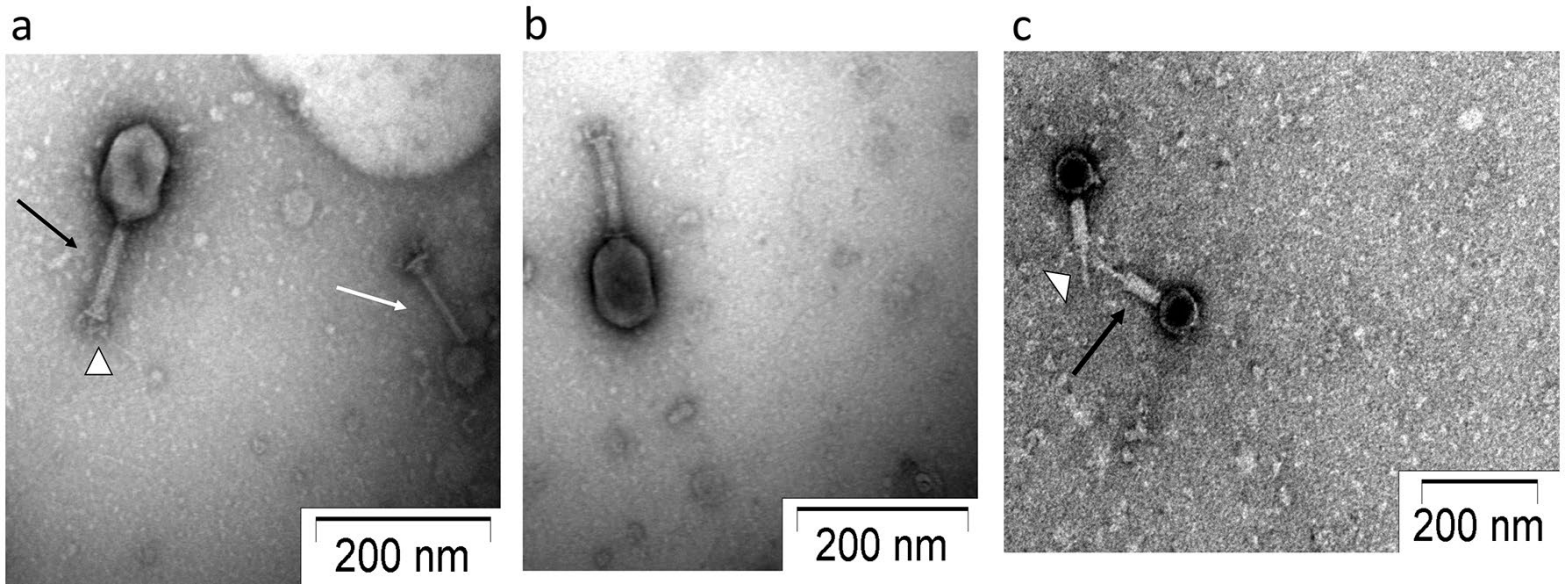
Transmission Electron micrographs of negatively stained phage particles from different areas on a grid. (**a**,**b**) Shows intact *Myovirida*e phage particles with uncontracted tail (black arrow) and base plate (arrowhead), broken tail (white arrow). (**c**) Phage particles with contracted tail sheath (black arrow) and protrusion of tail tubes (arrowhead). Purified phage particles were negatively stained using 2% (w/v) uranyl acetate and visualized using a TEM operating at a voltage of 100 kV.

Isolated phage was initially screened against *P. aeruginosa* through spot tests. A clear zone over a bacterial lawn was observed due to the lytic activity of the phage (Fig. 1a). This phage was named ‘DRL-P1’. On double-layer agar (DLA) plates, DRL-P1 produced small but clear plaques of approximately 2 ± 0.23 mm diameter (*n* = 56) of similar morphology (Fig. 1b). For subsequent characterization, bacteriophage enrichment was performed by repeated plaque purification method and a stock of 10^9^ PFU mL^−1^ was prepared (Fig. 1c). Purified phage particles were examined under a transmission electron microscope (TEM) and following International Committee on Taxonomy of Viruses (ICTV) guidelines, classified as a member of the bacteriophage family *Myoviridae*, signified by a head, neck, contractile tail, base plate, tail fiber geometry (Fig. 2). The average (*n* = 9) particle length (head to the base plate), head diameter, head length, tail length, tail diameter, and base-plate diameter was measured to be 197.47 ± 1.72, 68.89 ± 2.37, 93.03 ± 2.85, 94.54 ± 2.52, 16.82 ± 1.80, and 27.92 ± 3.03 nm, respectively.

### Antibiotic sensitivity and host range

The antibiotic response pattern of the bacterial isolate used in this study is tabulated as supplementary data (Table S1). The isolate showed resistance against Ceftazidime (CAZ), Nitrofurantoin (NIT), Nalidixic acid (NA), Ampicillin (AMP), Co-Trimoxazole (COT). However, it was found to be sensitive to Ciprofloxacin (CIP), Amikacin (AK), Amoxyclav (AMC), Cefotaxime (CTX), and Gentamicin (GEN), while intermediate sensitivity was noted against Netillin (NET), Tobramycin (TOB).

The host range of the phage DRL-P1 was examined against various standard bacterial cultures, obtained from the Microbial Type Culture Collection and Gene Bank (MTCC, Institute of Microbial Technology, Chandigarh, India). Phage DRL-P1 did not show any lytic activity against *Escherichia coli* (MTCC 443), *Vibrio cholera* (MTCC 3904), *Bacillus megaterium* (MTCC 428), *Shigella flexneri* (MTCC 1457), *Bacillus subtilis* (MTCC 1305), *Salmonella enterica* Typhimurium (MTCC 1251, MTCC 1252), *Streptococcus pyogene*s (MTCC 442) and *Klebsiella pneumoniae* (MTCC 8911). However, DRL-P1 showed clear lytic activity against *P. aeruginosa* (MTCC 1688) and also against nine other *P. aeruginosa* isolates (IS1-IS9), field-collected from the soil of Arunachal Pradesh, a northeastern state of India (Supplementary data, Table S2).

### Features of the DRL-P1 genome

Next-Generation Sequencing of the phage DNA resulted in the generation of a total of 1,295,948 raw reads (read length 150) amounting to 194.4 Mb bases. After sequence QC, a total of 1,221,536 reads (178.93 Mb bases) were used to assemble a terminally redundant genome of 66,243 nts having GC content of 54.9%, consisting of 22.75% A, 22.31% T, 27.52% G, and 27.40% C. The genome sequence was predicted to be ‘*intact*’ (completeness score 120) in the PHASTER analysis. In Blastn (Megablast) search, the DRL-P1 genome sequence was found to be significantly similar (up to 97.77% nucleotide identity over 99% query coverage) to Pseudomonasphage genome sequences belonging to the genus *Pbunavirus* (Order *Caudovirales*; Family *Myoviridae*), with top 10 hits namely being isolates- ‘DL52’ (KR054028), ‘misfit’ (MT119367), ‘zikora’ (MW557846), ‘R26’ (NC_048663), ‘datas’ (NC_050143), ‘Epa 14’ (NC_050144), ‘billy’ (MT133563), ‘elmo’ (MT119364), ‘kraken’ (KT372692), ‘Jollyroger’ (KT372691).

**Figure 3:**
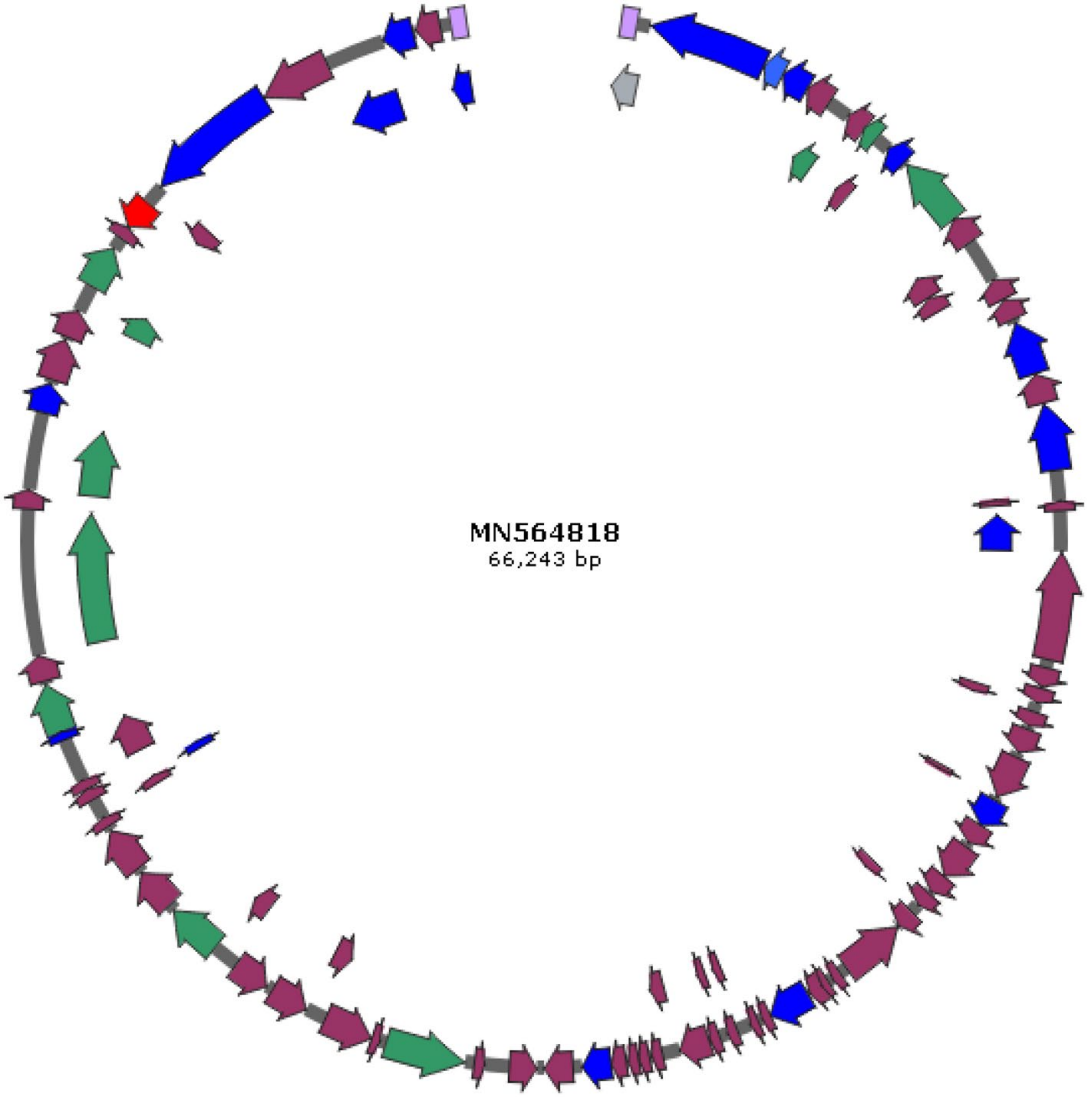
Complete genome map of 66,243 bp DRL-P1 dsDNA, visualized by SnapGene tool trial version (https://www.snapgene.com/). ORFs annotated by GeneMarkS and PHASTER servers are represented as arrows, where the arrowheads denote orientation of the respective ORFs. ORFs encoding structural, functional, lytic and hypothetical genes are shown by blue-, green-, red-, and maroon-colored arrows, respectively. The two terminal repeats are shown by lavender boxes. Details of the ORFs are presented in the annotation Table 1.

A total of 93 phage-hit ORFs were identified in the genome, of which 36 were functionally annotated based on homology with similar phage proteins available in the databases, while 57 were annotated as phage hypothetical proteins. Predicted ORFs were found to encode proteins ranging from 31 to 1035 aa in length, the largest being the DNA polymerase (Table 1). Identified ORFs included genetic regions, responsible for encoding proteins related to virion structure, genome replication, assembly & packaging, DNA synthesis & repair, regulation of gene expression, host identification & infection, host lysis, and recombination that are essential for the phage cycle. Among the 93 ORFs, 54 (58%) and 39 (42%) ORFs were encoded on each of the strands of the dsDNA, respectively. The strand with most of the ORFs was considered as the plus strand in further analyses. A genome map showing predicted ORFs (with definite phage-related proteins) is presented in (Fig. 3). Together, all the ORFs were encoded within 65,495 bps (from nts 634 to 66,128), resulting in an extremely high coding density of 98.87%. Notably, the start codon of 25 ORFs (26.88%) overlapped with the stop codon of the previous gene, suggesting transcriptional interactions among these neighboring genes. No putative tRNA encoding gene was identified in the genome. A total of 83 promoter regions and 27 Rho-independent terminators across the genome were predicted (Supplementary data, Tables S3 and S4). No toxin or antimicrobial resistance-related gene was predicted in the genome. Despite *in-silico* prediction as a temperate phage (averaged probability ± SD, 0.534 ± 0.03), DRL-P1 always presented highly lytic activity in in vitro assays.

**Table 1:** Predicted ORFs, their positions on the DRL-P1 genome, size, annotations and probable role in phage life cycle.

REGIONs/ORFs	Span		Strand	Length		GenBankannotation	Role/function in phage life cycle
	Start	Stop		ORF	aa		
REGION 1	1	353	+	353		Terminal repeat	Genome replication & packaging
ORF1	634	3216	−	2583	860	Phage internal (Core) protein	Virion Structure
ORF2	3220	3648	−	429	142	Phage exonuclease	Genome replication
ORF3	3658	4251	−	594	197	Phage tail fiber protein	Host infection
ORF4	4260	4799	−	540	179	Hypothetical protein	
ORF5	4799	5302	−	504	167	Phage tail fiber protein	Host infection
ORF6	5312	5740	−	429	142	Hypothetical protein	
ORF7	5742	6092	−	351	116	Phage exonuclease	Replication
ORF8	6089	6412	−	324	107	Hypothetical protein	
ORF9	6412	6864	−	453	150	Phage tail fiber Protein	Host infection
ORF10	6922	8436	−	1515	504	Putative transcriptional regulator	Gene expression
ORF11	8453	9034	−	582	193	Hypothetical protein	
ORF12	9031	9582	−	552	183	Hypothetical protein	
ORF13	9590	9988	−	399	132	Hypothetical protein	
ORF14	9985	10,452	−	468	155	Hypothetical protein	
ORF15	10,467	10,904	−	438	145	Hypothetical protein	
ORF16	11,006	12,154	−	1149	382	Phage capsid and scaffold Protein	Virion assembly
ORF17	12,164	12,799	−	636	211	Hypothetical protein	
ORF18	12,803	14,230	−	1428	475	Phage capsid and Scaffold protein	Virion assembly
ORF19	14,743	14,883	−	141	46	Hypothetical protein	
ORF20	14,880	15,086	−	207	68	Hypothetical protein	
ORF21	15,106	15,942	−	837	278	Phage minor capsid protein	Virion Structure & assembly
ORF22	15,942	18,239	−	2298	765	putative minor head protein	Virion Structure
ORF23	18,419	18,820	+	402	133	Hypothetical protein	
ORF24	18,852	19,175	+	324	107	Hypothetical protein	
ORF25	19,172	19,375	+	204	67	Hypothetical protein	
ORF26	19,381	19,692	+	312	103	Hypothetical protein	
ORF27	19,776	20,288	+	513	170	Hypothetical protein	
ORF28	20,392	21,324	+	933	310	Hypothetical protein	
ORF29	21,321	21,416	+	96	31	Hypothetical protein	
ORF30	21,426	22,019	+	594	197	Hypothetical protein	
ORF31	22,036	22,473	+	438	145	Hypothetical protein	
ORF32	22,559	23,338	+	780	259	Hypothetical protein	
ORF33	23,341	23,775	+	435	144	Hypothetical protein	
ORF34	23,820	24,170	+	351	116	Hypothetical protein	
ORF35	24,170	24,388	+	219	72	Hypothetical protein	
ORF36	24,385	24,768	+	384	127	Hypothetical protein	
ORF37	24,805	26,187	−	1383	460	Phage terminase, large subunit	DNA translocation and packaging termination
ORF38	26,387	26,575	+	189	62	Hypothetical protein	
ORF39	26,695	26,841	+	147	48	Hypothetical protein	
ORF40	26,852	27,154	+	303	100	Hypothetical protein	
ORF41	27,201	28,118	+	918	305	Phage tail length tape-measure protein	Genome injection
ORF42	28,121	28,309	+	189	62	Hypothetical protein	
ORF43	28,389	28,574	+	186	61	Hypothetical protein	
ORF44	28,699	28,899	+	201	66	Hypothetical protein	
ORF45	28,896	29,111	+	216	71	Hypothetical protein	
ORF46	29,108	29,299	+	192	63	Hypothetical protein	
ORF47	29,296	29,508	+	213	70	Hypothetical protein	
ORF48	29,536	30,183	+	648	215	Hypothetical protein	
ORF49	30,180	30,506	+	327	108	Putative single-stranded DNA binding protein	Genome replication
ORF50	30,572	30,796	+	225	74	Hypothetical protein	
ORF51	30,850	31,077	+	228	75	Phage dihydrofolate reductase	DNA synthesis
ORF52	31,087	31,308	+	222	73	Hypothetical protein	
ORF53	31,356	31,673	+	318	105	Phage single-stranded-DNA-specific exonuclease	Genome replication
ORF54	31,683	32,309	+	627	208	Phage putative head protein	Virion Structure
ORF55	32,502	33,116	+	615	204	Hypothetical protein	
ORF56	33,279	33,857	−	579	192	Hypothetical protein	
ORF57	34,388	34,618	−	231	76	Hypothetical protein	
ORF58	34,817	36,547	−	1731	576	Phage-associated DNA primase	Genome replication
ORF59	36,695	36,880	−	186	61	Hypothetical protein	
ORF60	36,886	37,962	−	1077	358	Hypothetical protein	
ORF61	37,959	38,408	−	450	149	Hypothetical protein	
ORF62	38,408	39,250	−	843	280	Hypothetical protein	
ORF63	39,381	40,166	−	786	261	Hypothetical protein	
ORF64	40,334	40,756	+	423	140	Hypothetical protein	
ORF65	40,743	41,930	+	1188	395	Capsid decoration protein	Virion Structure
ORF66	42,092	42,982	+	891	296	Hypothetical protein	
ORF67	43,087	44,088	+	1002	333	Hypothetical protein	
ORF68	44,178	44,408	+	231	76	Hypothetical protein	
ORF69	44,408	44,626	+	219	72	Hypothetical protein	
ORF70	44,610	44,828	+	219	72	Phage tail assembly protein	Virion Structure
ORF71	44,828	45,094	+	267	88	Hypothetical protein	
ORF72	45,106	45,312	+	207	68	Phage minor tail protein	Virion Structure
ORF73	45,312	46,229	+	918	305	Thymidylate synthase	DNA synthesis
ORF74	46,231	46,422	+	192	63	Phage tail assembly protein	Virion Structure
ORF75	46,425	47,465	+	1041	346	5'Polynucleotide kinase-3'phosphatase	DNA damage repair
ORF76	47,541	48,095	+	555	184	Hypothetical protein	
ORF77	48,095	51,202	+	3108	1035	Phage DNA polymerase III alpha subunit	Genome replication
ORF78	51,195	51,605	+	411	136	Phage Recombination protein	General recombination
ORF79	51,602	53,161	+	1560	519	Phage DNA Helicase	Genome replication
ORF80	53,256	53,876	+	621	206	Phage tail fiber protein	Virion Structure / Host infection
ORF81	53,965	54,861	+	897	298	Hypothetical protein	
ORF82	54,917	55,522	+	606	201	Hypothetical protein	
ORF83	55,519	56,073	+	555	184	Phage DNA Binding protein	Genome replication
ORF84	56,127	57,038	+	912	303	Phage DNA Ligase	Genome replication
ORF85	57,318	57,569	+	252	83	Hypothetical protein	
ORF86	57,594	58,256	−	663	220	Phage endolysin	Host cell Lysis
ORF87	58,256	58,684	−	429	142	Phage tail fiber component	Virion Structure
ORF88	58,687	61,581	−	2895	964	Phage tail fiber protein	Virion Structure
ORF89	61,586	63,100	−	1515	504	Hypothetical protein	
ORF90	63,097	64,350	−	1254	417	Phage tail assembly protein	Virion Structure
ORF91	64,407	65,072	−	666	221	Baseplate protein	Virion Structure
ORF92	65,128	65,661	−	534	177	Hypothetical protein	
ORF93	65,661	66,128	−	468	155	Phage minor tail protein	Virion Structure
REGION2	65,891	66,243	+	353		Terminal Repeat	Genome replication & packaging

**Figure 4:**
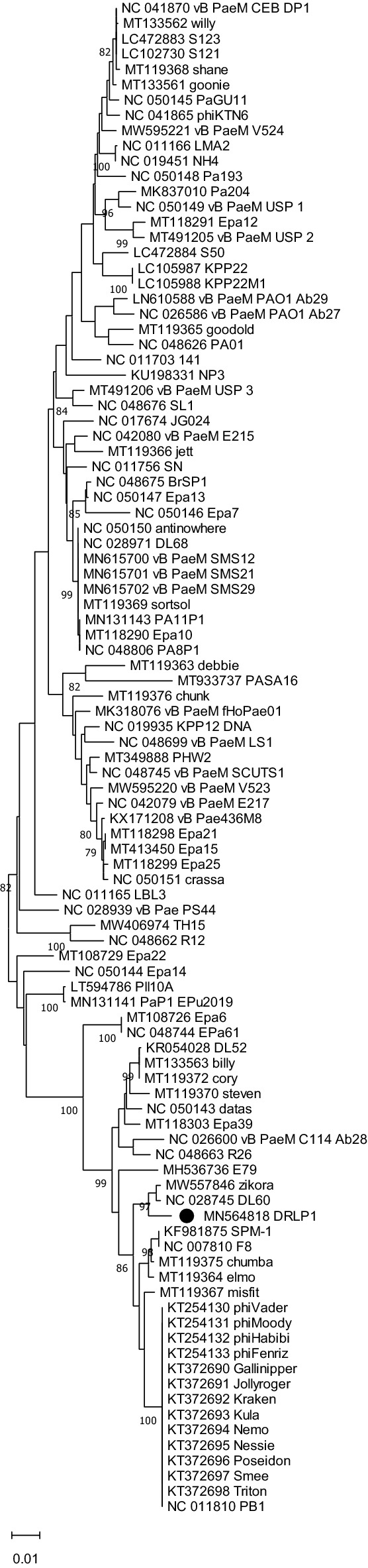
Neighbor-Joining phylogenetic tree based on the large terminase gene sequences from DRL-P1 and related 98 GenBank sequences. Multiple sequences were aligned using the MAFFT online server. Evolutionary distances were calculated using the Maximum Composite Likelihood method implemented in MEGA X computer program. Numbers below the branches represent percentage of replicate trees, where the associated taxa clustered together during the bootstrap test (1000 replicates). The optimal tree is drawn to scale with branch lengths signifying the evolutionary distances used to infer the tree. A total of 1383 positions were available in the final dataset for evolutionary analyses. Ambiguous positions were excluded from analysis. Graphical presentation of the phylogeny was generated using the MEGA X computer program, version 10.2.4 (https://www.megasoftware.net/).

**Figure 5:**
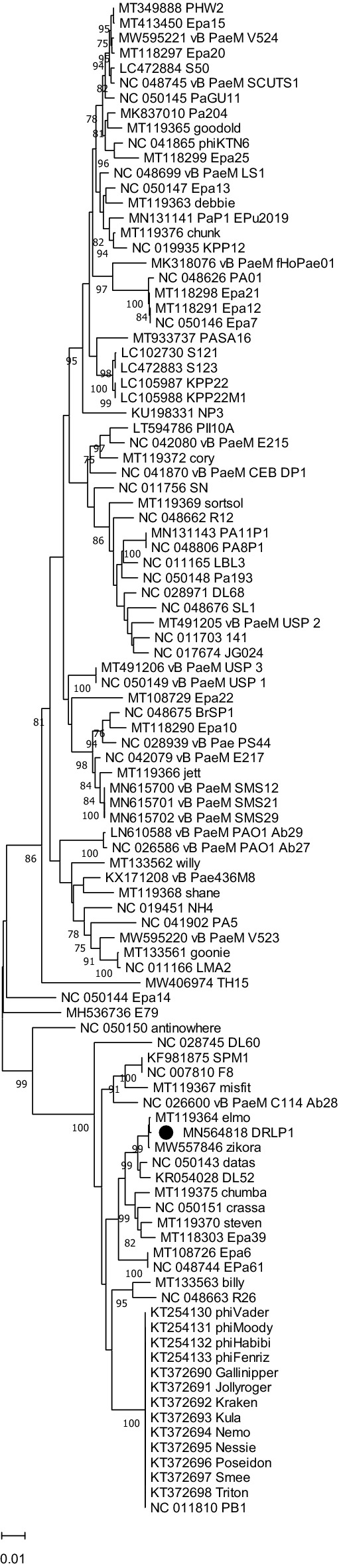
Neighbor-Joining phylogenetic tree based on the DNA pol III sequences from DRL-P1 and related 100 GenBank sequences. Multiple sequences were aligned using the MAFFT online server. Evolutionary distances were calculated using the Maximum Composite Likelihood method implemented in MEGA X computer program. Numbers below the branches represent percentage of replicate trees, where the associated taxa clustered together during the bootstrap test (1000 replicates). The optimal tree is drawn to scale with branch lengths signifying the evolutionary distances used to infer the tree. A total of 3111 positions were available in the final dataset for evolutionary analyses. Ambiguous positions were excluded from analysis. Graphical presentation of the phylogeny was generated using the MEGA X computer program, version 10.2.4 (https://www.megasoftware.net/).

**Figure 6:**
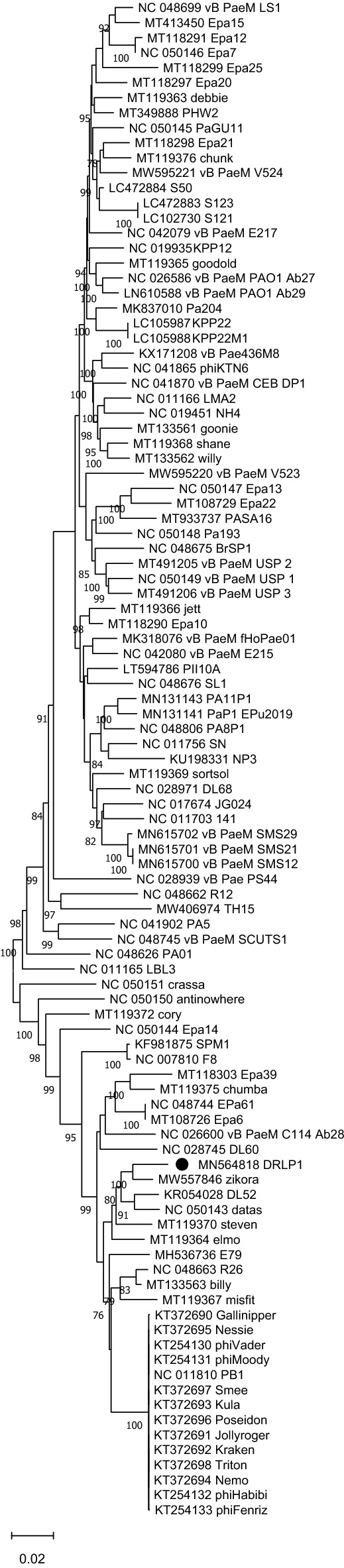
Neighbor-Joining phylogenetic tree based on complete genome sequences from DRL-P1 and related 100 GenBank sequences. Multiple sequences were aligned using the MAFFT online server. Evolutionary distances were calculated using the Maximum Composite Likelihood method implemented in MEGA X computer program. Numbers below the branches represent percentage of replicate trees, where the associated taxa clustered together during the bootstrap test (1000 replicates). The optimal tree is drawn to scale with branch lengths signifying the evolutionary distances used to infer the tree. A total of 147,417 positions were available in the final dataset for evolutionary analyses. Ambiguous positions were excluded from analysis. Graphical presentation of the phylogeny was generated using the MEGA X computer program, version 10.2.4 (https://www.megasoftware.net/).

Neighbor-Joining (NJ) phylogenetic trees were reconstructed for terminase and DNA polymerase III genetic sequences with top 100 BLAST hit sequences (including RefSeq sequences). In the terminase sequence phylogeny (Fig. 4), DRL-P1 clustered most closely with a *Pbunavirus* RefSeq sequence ‘DL60’ (NC_028745), and an unclassified *Pbunavirus* sequence 'zikora' (MW557846), having a divergence of 0.0124 base substitutions per site with both the sequences. Conversely, in the DNA polymerase III sequence phylogeny (Fig. 5), DRL-P1 clustered most closely with two unclassified *Pbunaviruses*, ‘zikora’ and ‘elmo’ (MT119364), both showing the divergence of 0.001 base substitutions per site, followed by close affiliation to RefSeq *Pbunavirus* 'datas’ (NC_050143) and ‘DL52’ (KR054028) having divergences of 0.0068 and 0.0133 base substitutions per site, respectively. Similar to the DNA polymerase III gene sequence phylogeny, in the complete genome phylogeny, DRL-P1 clustered with *Pbunavirus* isolates 'zikora', ‘DL52’, ‘datas’, ‘steven’ (MT119370), and 'elmo’, supported by high bootstrap values (Fig. 6).

**Figure 7:**
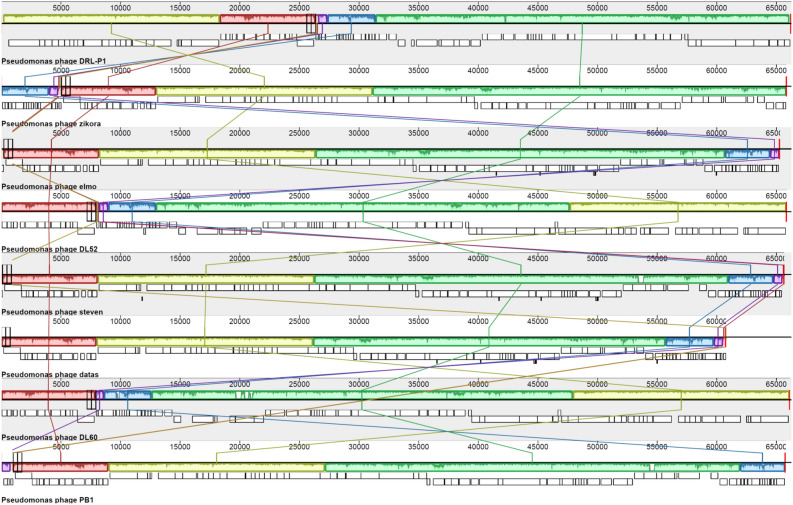
Results of Mauve progressive alignment of annotated complete genome sequences of DRL-P1 with related *Pbunaviruses* ‘zikora’, ‘elmo’, ‘DL52’, ‘steven’, ‘datas’, ‘DL60’ and ‘PB1’ (from top to bottom). Relative position of the homologous regions or the locally collinear blocks (LCBs) shared by two or more genomes are depicted by same colors. Similarity of the LCBs between genomes are signified by plot within the blocks, where the height of the plot represents mean nucleotide identity. Relative position of the homologous LCBs among the genomes are indicated by thin vertical lines of same-color. The white blocks under the LCBs represent genome features (annotated ORFs) obtained from the GenBank. Graphical presentation of the alignment of LCBs was generated using the progressiveMauve computer program version 2.4.0 (http://darlinglab.org/mauve/mauve.html).

To further elucidate the taxonomic position of DRL-P1, we used the VIRIDIC program to calculate pairwise intergenomic similarities between DRL-P1 and 100 top BLAST hit *Pbunavirus* genomes including 37 RefSeq and 63 other complete genome sequences retrieved from the GenBank. In concurrence with the complete genome phylogeny, the VIRIDIC program clustered DRL-P1 with ‘DL52’, ‘zikora’, ‘elmo’, and ‘steven’ as a separate species under the genus *Pbunavirus* (Supplementary data, Tables S5,S6), presenting with 96.0% to 97.5% nucleotide identity among themselves. On the other hand, DRL-P1 showed 93.3% and 92.8% nucleotide identity with *Pbunavirus* RefSeq sequences ‘DL60’ and ‘datas’, respectively, which were suggested as close relatives in the phylogenetic analysis of the terminase and DNA polymerase III genetic regions. Although DRL-P1 showed high intergenomic similarities and close phylogenetic relatedness to these phage genomes isolated from different parts of the world, results of progressive multiple genome alignment analysis demonstrated that the arrangement of locally colinear blocks (LCBs) in the DRL-P1 genome was substantially distinct (Fig. 7).

**Figure 8:**
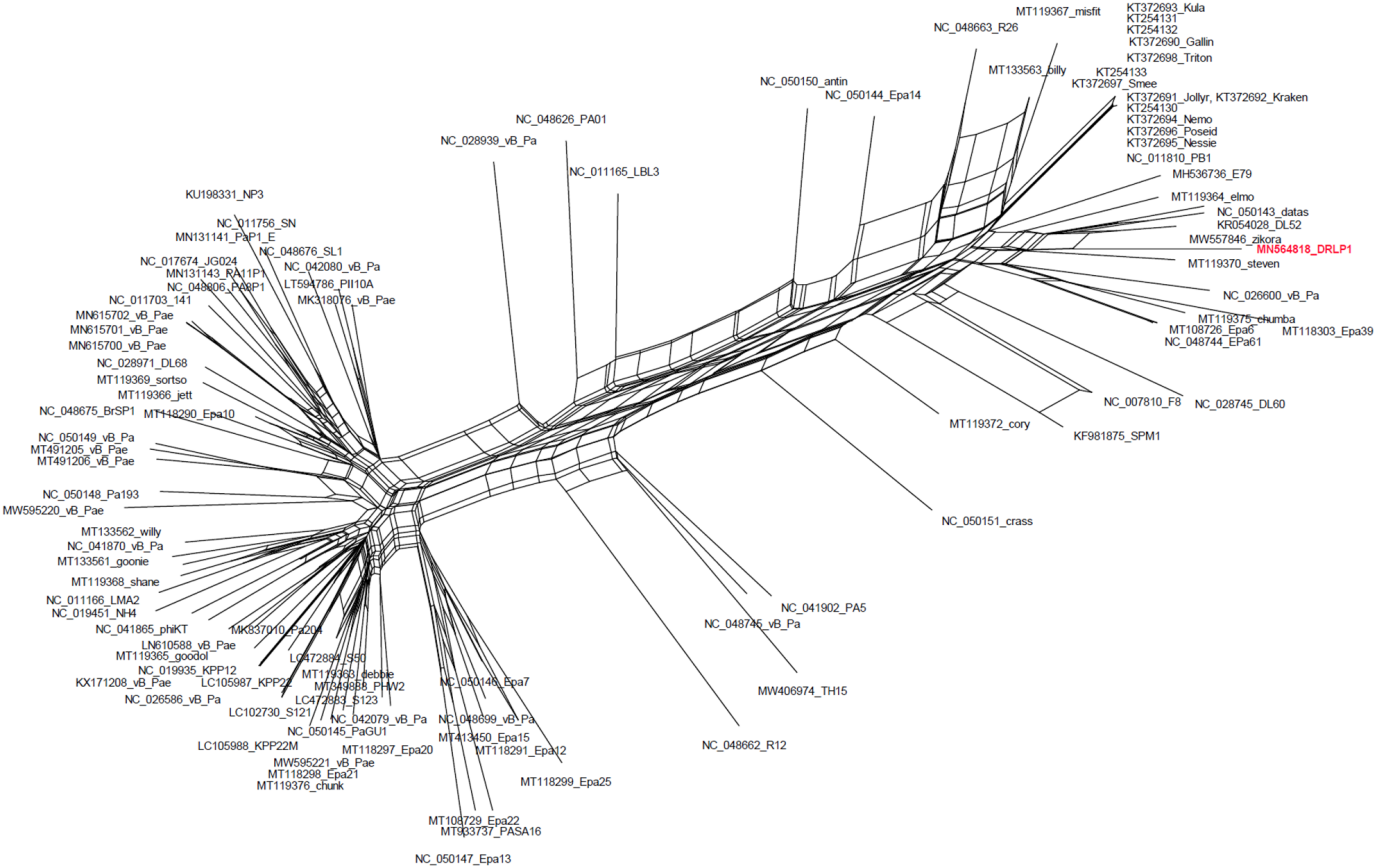
Neighbor-Net network tree was reconstructed with 100 GenBank sequences including 37 NCBI-RefSeq and DRL-P1complete genome sequence, using the SplitsTree4 computer program. Sequences were aligned using the CLUSTALW program. Kimura-2 parameter algorithm was employed for estimating genetic differences. The dataset included a total of 119,869 positions. Gaps and parsimony uninformative sites were excluded from analysis. Graphical presentation of the network tree was generated using SplitsTree4 computer program (version 4.14.6; https://uni-tuebingen.de/en/fakultaeten/mathematisch-naturwissenschaftliche-fakultaet/fachbereiche/informatik/lehrstuehle/algorithms-in-bioinformatics/software/splitstree/).

**Figure 9:**
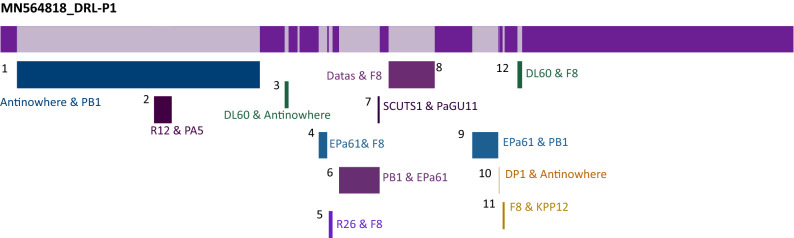
Recombination map showing 12 recombination events in the DRL-P1 genome, as detected by the RDP4 computer program. The dataset included DRL-P1 and 37 NCBI-RefSeq complete genome sequences. Details of the recombination events are presented in Table 2. Minor and Major parent involved in each of the recombination event is indicated by the most similar RefSeq isolate name. Graphical presentation of the recombination events was generated using the RDP4 computer program (version 4.95; http://web.cbio.uct.ac.za/~darren/rdp.html).

Minor differences in terminase and DNA polymerase III phylogenetic tree topologies along with the observed differences in the arrangement of LCBs in the DRL-P1 genome as compared to closely related *Pbunavirus* genomes indicated the possibility of horizontal gene transfer or recombination. Therefore, a NN was reconstructed (Fig. 8) using the complete genome dataset previously used to reconstruct NJ phylogeny. The clustering pattern of sequences in the NN principally agreed to the NJ phylogeny, yet there were signals of genetic exchange in the form of extensive reticulation at the base of the DRL-P1 stock. The reticulation between DRL-P1 and other related genomes indicated multiple events of genetic exchanges, suggesting to rationalize the observed subtle divergences in terminase and the DNA polymerase III tree topologies. Subsequent analysis of recombination revealed the existence of genetic fragments similar to various other Pbunaviruses in the DRL-P1 genome, which seem to suggest the evolution of the DRL-P1 genome through the frequent exchange of genetic material (Fig. 9, Table 2).

**Table 2:** Details of recombination events detected in the DRL-P1 complete genome.

Events	Breakpoint positions (99% CI)		Parental sequences most similar to RefSeq		Detection method						
	Begin	End	Minor parent	Major parent	RDP	GENECONV	BOOTSCAN	MAXCHI	CHIMAERA	SISSCAN	3SEQ
1	*1–2508	27,450–27,676	NC_050150_antinowhere	NC_011810_PB1	NS	7.63E−05	1.39E−05	3.44E−03	NS	2.33E−11	NS
2	12,952–13,552	15,791–16,070	NC_048662_R12	NC_41902_PA5	3.87E−11	NS	9.28E−12	4.06E−14	5.68E−17	1.04E−21	NS
3	30,300–30,982	31,037–31,179	NC_028745_DL60	NC_050150_antinowhere	NS	6.07E−06	NS	2.13E−03	2.33E−04	9.07E−08	NS
4	34,741–34,864	35,766–36,483	NC_048744_EPa61	NC_007810_F8	2.17E−02	NS	4.30E−03	1.02E−09	7.24E−07	NS	7.76E−07
5	34,742–36,409	36,756–49,890	NC_048663_R26	NC-007810_F8	NS	4.52E−04	2.89E−06	3.05E−03	NS	NS	8.81E−05
6	37,757–39,593	40,875–41,707	NC_011810_PB1	NC_048744_EPa61	2.78E−03	4.94E−05	2.02E−02	7.31E−07	1.89E−05	5.20E−07	2.20E−19
7	40,875–41,089	41,394–41,482	NC_048745_SCUTS1	NC_050145_PaGU11	NS	4.24E−07	6.04E−18	5.99E−05	2.91E−04	2.45E−24	1.07E−06
8	42,697–42,783	49,313–50,275	NC_007810_F8	NC_050143_datas	1.81E−34	5.00E−59	3.02E−52	1.76E−22	3.73E−14	4.91E−33	1.32E−12
9	54,844–55,619	58,859–59,079	NC_048744_EPa61	NC_011810_PB1	4.78E−66	3.75E−88	1.38E−92	1.20E−37	1.13E−38	6.11E−48	NS
10	40,432–59,079	59,115–59,433	NC_041870_DP1	NC_050150_antinowhere	NS	3.31E−12	1.87E−12	2.63E−04	1.35E−04	NS	5.62E−11
11	59,386–59,605	59,938–60,186	NC_007810_F8	NC_019935_KPP12	5.51E−70	8.01E−73	1.85E−72	1.82E−13	6.12E−13	5.46E−24	5.62E−12
12	59,838–61,747	62,466–62,776	NC_028745_DL60	NC_007810_F8	NS	NS	2.87E−05	4.85E−09	1.85E−05	NS	3.74E−07

*NS* not significant.

### Phage adsorption and growth kinetics

**Figure 10:**
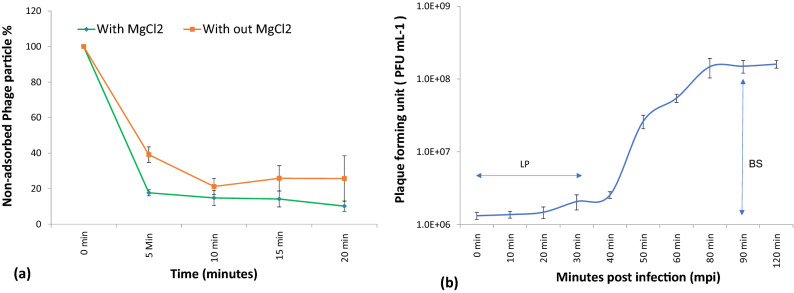
(**a**) Effect of magnesium ion on adsorption rate of *P. aeruginosa* bacteriophage (DRL-P1). (**b**) A single-step growth curve of phage DRL-P1 measured against *P. aeruginosa* at an MOI of 0.1. The growth curve suggests a latent phase (LP) of ~ 30 min, while a burst size (BS) of ~ 100 PFU per infected cell. The plots represent mean obtained from three independent replicate for each point and vertical whiskers represent SD. Plots were generated using the chart function incorporated in Excel program (MS Office version 18.2106.12410.0).

A maximum of 76% phage adsorption was documented within 20 min without any supplements. In comparison, when supplemented with MgCl_2_, approximately 82% of the phages were adsorbed within 5 min, while a maximum of 90% adsorption took place within 20 min. These results indicate a positive effect of Mg^2+^ ions on phage infectivity, probably by accelerating phage adsorption rate, thereby ensuring lysis of a maximum number of bacterial cells (Fig. 10a).

A single-step growth curve (Fig. 10b) was prepared to evaluate the latent period and the burst size of the phage DRL-P1. The latent period was found to be approximately 30 min which signifies the time interval between phage adsorption and the start of the first burst. On the other hand, the duration of the rise period was estimated to be around 50 min with a burst size of approximately 100 PFU per infected cell was recorded in the experiments.

### Stability of phage at different temperatures and pH conditions

**Figure 11:**
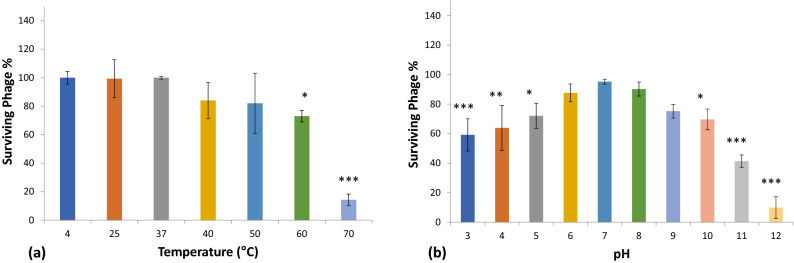
Stability of phage DRL-P1 under different thermal and pH conditions. (**a**) Graph showing effects of different temperature conditions on the stability of DRL-P1. Phage aliquots were incubated at different temperatures, 25 °C, 37 °C, 40 °C, 50 °C, 60 °C and 70 °C for 60 min, followed by enumeration of viable phages by standard double-layer plaque assay. Stability of phage stored at 4 °C was considered as control for comparison. Data obtained from three independent experiments are represented here as mean ± SD. ***P < 0.001 or *P < 0.05 indicates a significant reduction at temperature 60 °C & 70 °C in comparison to initial PFU count. (**b**) Graph showing effects of different pH conditions on the stability of phage DRL-P1. Aliquots of phage were added to buffers adjusted to various pH conditions (1.0–14.0), incubated at room temperature for 18 h, followed by enumeration of viable phages by standard double-layer plaque assay. Data obtained from three independent experiments are represented here as mean ± SD. ***P < 0.001, **P < 0.01 or *P < 0.05 indicates a significant reduction level at pH 3, 4, 5, 10, 11 &12 in comparison to initial PFU count (as compared to phage stored in TM buffer pH 7.4 at 4 °C). Plots were generated using the chart function incorporated in Excel program (MS Office version 18.2106.12410.0) and statistical analysis were performed using the GraphPad PRISM computer program (Trial version 7.05; https://www.graphpad.com/scientific-software/prism/).

The temperature vs. phage stability was studied at six different temperatures viz. 25 °C, 37 °C, 40 °C, 50 °C, 60 °C, and 70 °C. Storage at 4 °C was considered as the control for the temperature stability experiments (Fig. 11a). Our results demonstrated that the phage DRL-P1 was substantially stable at 25 °C, 37 °C temperatures, while moderately stable at 40 °C and 50 °C temperatures. However, at a temperature of 60 °C and above, phage stability decreased significantly. Specifically, as compared to control, 72% of the phages survived 60 °C temperature (*P < 0.05), whereas, only 14% of the phages could survive 70 °C temperature (***P < 0.001), after the experiment.

Our study demonstrated that after 18 h of incubation under different pH conditions, phage DRL-P1 was most stable at pH 6.0, 7.0, and 8.0, and no significant loss in the titer was observed. However, below pH 3.0 and beyond pH 10.0, only minute fractions of the viable phages were found. Approximately, 70% of the phage population was viable between pH 5.0 to 10.0. As compared to the initial titer, a significant reduction resulting in the survival of 65% and 72% phages was observed at pH 4.0 (**P < 0.01) and pH 5.0 (*P < 0.05), respectively. No viable phages were recovered after incubation at extreme pH 1.0, 2.0, 13.0, and 14.0 conditions (Fig. 11b).

### Decontamination of fomites through phage preparations

**Figure 12:**
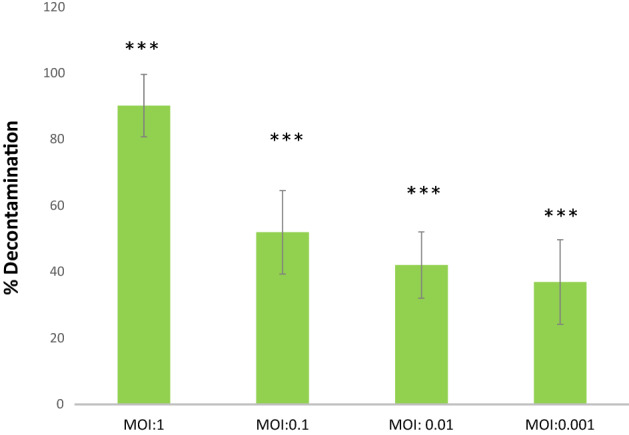
Graphical representation of results from decontamination assay experiments using artificially contaminated cover-slip model for contaminated surfaces. Decontamination of artificially contaminated glass cover slip with phage DRL-P1 application at different MOI (1.0, 0.1, .01, .001). MOI:0 represents control (no phage treatment). Bars represent average % reduction of *P. aeruginosa* after phage treatment at different MOIs. Data represents mean ± SD from the triplicate experiments. ***P < 0.001 indicates a significant difference between phage treatment at different MOI and the control with no phage treatment. Plots were generated using the chart function incorporated in Excel program (MS Office version 18.2106.12410.0) and statistical analysis were performed using the GraphPad PRISM computer program (Trial version 7.05; https://www.graphpad.com/scientific-software/prism/).

In the present work, a glass coverslip was used to represent the contaminated solid surface. The decontamination potential of the DRL-P1 phage was determined at different MOIs. A 90% reduction in the bacterial count was recorded at MOI:1.0 (***P < 0.001), whereas at MOI:0.1, 52% (***P < 0.001) reduction was observed. As compared to no-phage control, a significant (***P < 0.001) reduction in the bacterial count was recorded at even low MOIs of 0.01 and 0.001, demonstrating 42% and 37% reduction, respectively. Data (mean ± SD) obtained from the experiments are represented in Fig. 12.

### Phage action on bacteria

**Figure 13:**
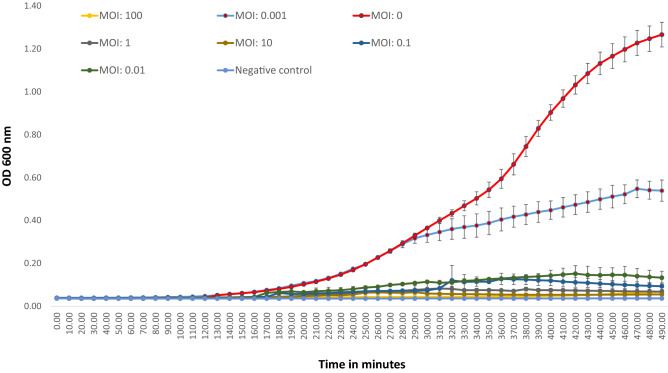
In vitro lytic activity of DRL-P1 against *P. aeruginosa.* Lytic activity was studied at Multiplicity of Infection (MOI), 100, 10, 1, 0.1, 0.01, 0.001 for 8 h. Bacterial growth was recorded by changes in absorbance (OD_600_) using an automated multi-mode plate reader. Data displayed in the plot represent mean ± SD of three independent experiments. Plots were generated using the chart function incorporated in Excel program (MS Office version 18.2106.12410.0).

Phage action on bacterial growth was observed through a change in OD_600_ over 8 h of incubation. Data from the no-phage control experiment (MOI:0) showed a typical sigmoid curve representing an uninhibited bacterial growth, whereas experiments set up with different MOIs (100, 10, 1, 0.1, 0.01, and 0.001) indicated inhibition of bacterial growth due to phage action (Fig. 13).

### Biofilm degradation assay

**Figure 14:**
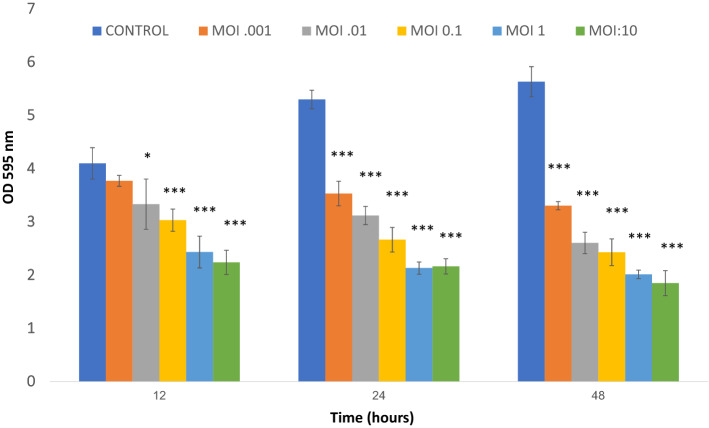
Graph showing the results of DRL-P1 mediated *P. aeruginosa* biofilm degradation assay performed at various MOIs (10, 1, 0.1, 0.01, 0.001). Non-phage treated biofilm was used as a positive control. OD at 595 nm was measured at 12, 24 and 48 h. Data represents mean ± SD from the triplicate experiments. ***P < 0.001 or *P < 0.05 indicate significant difference between the control and the respective phage treated samples after a particular period of time. Plots were generated using the chart function incorporated in Excel program (MS Office version 18.2106.12410.0) and statistical analysis were performed using the GraphPad PRISM computer program (Trial version 7.05; https://www.graphpad.com/scientific-software/prism/).

In our experiments, DRL-P1 showed degradation potential against pre-established *P. aeruginosa* biofilms (Fig. 14). Using the standard method of phage-bacteria co-cultures set-up at various MOIs, we could observe a considerable decrease in bacterial biomass after 12, 24, and 48 h of incubation. In co-cultures set-up at MOI:10, significant loss (***P < 0.001) of approximately 45%, 59%, and 67% biomass were observed after 12 h, 24 h, and 48 h incubation, respectively. Similarly, at a MOI:1.0, significant (***P < 0.001) loss of approximately 40%, 59%, and 64% biomass were observed after 12 h, 24 h, and 48 h incubation, respectively, as compared to the untreated phage control. DRL-P1 also exhibited a significant biofilm degradation efficiency even at relatively lower MOIs 0.01, 0.001 (***P < 0.001 and ***P < 0.001, respectively) after 24 and 48 h of incubation (Fig. 14).

### Stability of phage after lyophilization and encapsulation within alginate

**Figure 15:**
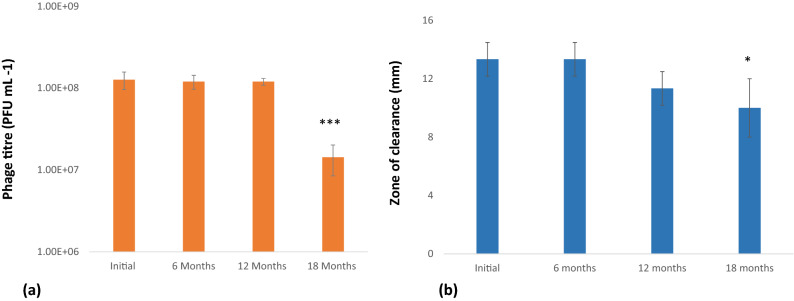
(**a**) Stability of lyophilized DRL-P1 at different time interval. Lyophilized phages were reconstituted in TM buffer and titers determined through standard double layer plaque assay. Bars represents mean ± SD from the three vials tested at each time point. ***P < 0.001 indicate significant difference between the initial titre and the stored lyophilized phage samples, after 18 months. (**b**) Stability of DRL-P1 in alginate capsules at different time interval. Zone of clearance by phage loaded alginate beads over the lawn of *P. aeruginosa* was measured. Bars represents mean ± SD from the three samples tested at each time point. *P < 0.05 indicate significant difference between the initial titre and the phage loaded in alginate beads after 18 months. Plots were generated using the chart function incorporated in Excel program (MS Office version 18.2106.12410.0) and statistical analysis were performed using the GraphPad PRISM computer program (Trial version 7.05; https://www.graphpad.com/scientific-software/prism/).

Lyophilization of bacteriophage (~ 10^9^ PFU mL^−1^) resulted in an initial drop in the phage titer (~ 10^8^ PFU mL^−1^). For experiments, lyophilized phages were reconstituted in 2 mL TM buffer and plaque assay was performed to determine PFU. Our results indicate that once lyophilized, DRL-P1 retained its lytic activity without any significant drop in titer up to 12 months. However, after 18 months of storage (tested so far), the PFU of the lyophilized sample was found to be significantly reduced (~ 10^7^ PFU mL^−1^, ***P < 0.001) (Fig. 15a).

The lytic activity of phage-loaded and mock-loaded (only TM buffer) alginate beads (5–6 mm) was tested by placing the beads over a lawn of *P. aeruginosa*. A clear zone was measured to determine the lytic potential of the DRL-P1 phage loaded within the alginate beads. Each of the freshly prepared phage-loaded beads showed ~ 13 ± 1.1 mm zones of clearance. No statistically significant change in the zone of clearance was noted in assays performed with phage-loaded beads stored for 6 months and 12 months, showing ~ 13 ± 1.0 and ~ 11 ± 1.1 mm zones of clearance, respectively. However, after 18 months of storage, the beads showed a significant decrease (*P < 0.05) in a zone of clearance (~ 10 ± 2 mm) (Fig. 15b).

## Discussion

In India, more than 50% of the *P. aeruginosa* isolates are reported to be resistant to broad-spectrum antibacterial (fluoroquinolones) and third-generation beta-lactam antibiotic (cephalosporins)^5,6^. The *P. aeruginosa* isolate used in the present study was resistant to multiple antibiotics including Ceftazidime (a beta-lactam, 3rd-gen bactericidal cephalosporin antibiotic), Ampicillin (a semi-synthetic beta-lactam, broad-spectrum bactericidal), Nalidixic acid (a synthetic quinolone, antibacterial), Nitrofurantoin (a synthetic derivative of imidazolidinedione, antibiotic) and Co-Trimoxazole (a combination of trimethoprim and sulfamethoxazole), which are widely used in the treatment of bacterial infections involving multiple organs^27^. Interestingly, aggregates of *P. aeruginosa* can encase themselves within biofilms through the synthesis of extracellular matrix, making them almost impossible to treat or eradicate with antibiotics^15^. Fortunately, phages are highly effective in the treatment of recalcitrant infections and removal of biofilms, thereby exposing the bacterial aggregates to the therapeutics^8,28–30^. Therefore, in the current scenario of infection with critical pathogens*,* phage therapy has emerged as a promising alternative to antibiotics, especially against biofilm-forming and multidrug resistance bacteria^15^.

Owing to the inherent advantages of phages over chemical antibacterials, several researchers have now focused their efforts on the isolation, characterization, and application of lytic phages against *P. aeruginosa* infections^13^. Although very few reports are available so far, Indian researchers have also endeavored upon attempts to isolate Pseudomonasphages. A PubMed literature search conducted on June 25th, 2021, using the query string “*bacteriophage* + *Pseudomonas* + *aeruginosa*” resulted in 1409 articles. On the other hand, a query string “*bacteriophage* + *Pseudomonas* + *aeruginosa* + *India*” resulted in 20 articles, of which, only 7 research articles reported the isolation and/or application of phages^19–25^. To the best of our knowledge, while the present manuscript was under revision, a group of researchers published a detailed characterization of a Pseudomonasphage (N4-like phage AM.P2, *Podoviridae* family), isolated from wastewater from the southern part of India^31^. In the present manuscript, we report the isolation of a lytic Pseudomonasphage DRL-P1 (*Myoviridae* family) from wastewater from the northeastern part of India and carried out its detailed characterization (growth parameters, TEM based morphology, whole-genome sequencing, analysis of toxin and antimicrobial genetic regions, etc.). Subsequently, we demonstrate the lytic potential of DRL-P1 as a natural decontaminating agent.

We studied the growth parameters of DRL-P1 through a single-step growth curve which helps in defining phage lytic potential for biocontrol of bacteria^32,33^. Our results indicate a short latency period of approximately 30 min and a considerable burst size of approximately 100 phages per infected cell for DRL-P1. While the latency period defines the period between virion attachment to the host bacterium and the release of new phage particles, burst size defines the average number of new phage particles released from each infected cell after a lytic cycle^26^. Therefore, short latency and large burst size are considered characteristic features of an efficient lytic phage and indicate the suitability of a phage for therapeutic applications^26,34^. In addition, stability of the phages at various temperatures, pH conditions, and tolerance to storage techniques are also critical parameters for their practical application in diverse settings. In clinical applications, the candidate phage has to withstand different pH conditions depending upon the route of administration, while it has to tolerate a wide variation in pH and temperature in an environmental application^26,35^. Our data showing that DRL-P1 is substantially stable over a wide range of conditions including temperature, pH and can withstand techniques for storage, advocate the suitability of DRL-P1 for varied settings such as clinical, environmental, etc. Therefore, wastewater receiving sewage is considered to be one of the best sources for isolation of sturdy phages that have evolved to tolerate harsh and fluctuating physicochemical conditions.

A thorough analysis of the DRL-P1 complete genome revealed its identity as a member of the *Pbunavirus* genus under the family *Myoviridae* of tailed bacteriophages. Virus particle features, genome length, GC content, number of ORFs were similar to typical *Pbunaviruses*^36^. Despite Blast search showing close homology of DRL-P1 sequence with various *Pbunavirus* sequences submitted in the GenBank, molecular evolutionary analyses including the terminase and DNA polymerase III displayed minor differences in phylogenetic clustering (NJ trees), suggesting a non-homogenous evolutionary history (horizontal genetic exchange such as recombination) of the DRL-P1 genome. To further explore this possibility, we reconstructed reticulate evolutionary history (NN tree), which represents tree topologies more accurately in the case of datasets comprising sequences with recombination^37,38^. Based on observed reticulations in the NN trees, we performed a detailed analysis of recombination, which showed that the DRL-P1 genome is composed of genetic segments from different related Pbunaviruses. Bacteriophages, owing to their extremely distant origin (> 3 billion years ago), highly dynamic population structure and active engagement in exchange of genetic material, are shown to have ‘*pervasively mosaic*’ genomes^38^, which supports our findings of frequent recombination events in the DRL-P1 genome. Notably, high intergenomic similarity estimates and species clustering patterns obtained from the VIRIDIC analysis suggested that DRL-P1 along with at least four unclassified sequences submitted in the GenBank (zikora, elmo, steven, DL52) represents a distinct species cluster under the genus *Pbunavirus*. However, our MAUVE analysis results show that the arrangement of LCBs in the DRL-P1 genome is substantially distinct from the other phage genomes of this species cluster, isolated from widely distant parts of the world. Taken together, our findings point to an independent evolutionary history of DRL-P1 and thus advocate its classification as a separate taxon under the genus *Pbunavirus*, albeit the Bacterial and Archaeal Viruses Subcommittee of the ICTV has the final authority to review and ratify phage classifications.

It is well known that motility helps a bacterial species in colonizing new surfaces, spreading across the surface, and formation of biofilms^39^. *P. aeruginosa* is a highly motile bacterium, which uses flagellum-facilitated swimming to reach and attach surfaces, and spreads across the surface by flagella and type IV pili mediated swarming and twitching movements, respectively^39^. *P. aeruginosa* can even move by sliding in the absence of flagella and type IV pili and by surfing movement in response to stress^40,41^. Multiple modes of motility, quorum-sensing, ability to form resistant biofilms, multiple processes for adaptation, etc. make *P. aeruginosa* an ideal pathogen for nosocomial transmission through contaminated surfaces, invasive ventilation devices, urinary catheterization, nasogastric feeding devices, etc.^42^. Our results from decontamination assays showed significant efficiency of DRL-P1 in decontaminating solid surfaces at different MOIs. We here demonstrate that contaminated surfaces can be effectively decontaminated by phage treatment, which cannot be subjected to the standard methods of decontamination, such as exposure to UV, autoclaving, etc. Similar surface decontamination by phage application has earlier been shown by Jensen et al.^43^ and Rashid et al.^44^. Previous studies have also demonstrated effective phage-mediated control of *P. aeruginosa* biofilm formation on catheters/endotracheal tubes and control of biofilms in mono and mixed cultures using different in vitro and in vivo models^45–49^. Further, application of phage DRL-P1 at low MOI of 0.01 and 0.001 resulted in effective decontamination which indicates our phage as a promising decontaminating agent against *P. aeruginosa*.

In addition to laboratory characterization of the potential phages, their successful application requires stable preservation in suitable excipients or as formulations in pharmaceutical products. Despite its intrinsic significance in the practical application of phages, relatively fewer studies are available on the topic. Researchers are continuously trying to improve phage stability under different storage conditions. Lyophilization-based stabilization techniques are proposed to be suitable for inhalation formulations, storage, and transportation at non-refrigerated conditions^50^. On the other hand, polymer encapsulation-based phage formulations have been shown to protect the phages from harsh pH, digestive enzymes, bile juices in the gastrointestinal tracts, while facilitating permeability to mucous linings^51,52^. Recently, Manohar & Ramesh^50^ compared the effect of different excipients during phage lyophilization and reported that sucrose, gelatin, and their combination have beneficial effects on phage viability during long-term storage. In the present study, we observed that initially there was a drop in PFU due to the process of lyophilization, no significant decrease was noted afterward even after 12 months of storage. A similar decrease in phage titer due to lyophilization has been described by other researchers too^51,53,54^. We also attempted encapsulation of the phage DRL-P1 in alginate beads and have demonstrated retention of its lytic activity after encapsulation, similar to that reported by previous studies^52,55,56^. Taken together, our results of lyophilization and encapsulation studies with DRL-P1 demonstrate its compatibility with different preservation procedures for diverse applications.

Although in recent times, phage therapy has gained substantial momentum in the treatment of disease caused by *P. aeruginosa* and other bacterial infections, several challenges still exist in the wider application of phages for therapeutic and other applications. One of the primary challenges is our limited understanding of the *P. aeruginosa* defense mechanisms that bestow resistance against a single phage or cocktails and permit phage adaptation to the altering host systems^57^. On the other hand, using an in vitro lung co-culture enhanced microenvironment model, a recent study showed that certain Pseudomonasphage promotes the production of inflammatory cytokines IL-6 and TNF-α, which emphasize the importance of understanding immunological responses to phages for their successful application as therapeutics^58^. Additionally, a thorough examination of the phage genomes for the presence of toxin or antibiotic resistance-related genes, careful evaluation of safety associated with combined therapies (phage + antibiotics or other drugs), and development of appropriate methods to rapidly evaluate phage competence against uncharacterized clinical isolates or poorly differentiated *P. aeruginosa* clones^59^ are some of the challenge areas that have enormous scope for further research.

## Conclusion

The results of our phenotypic and genotypic characterization studies demonstrate that DRL-P1 is a virulent phage, belonging to the genus *Pbunavirus* under the *Myoviridae* family of bacteriophages. This phage was found to be highly lytic against MDR *P. aeruginosa*. DRL-P1 features short adsorption time, large burst size, stability over a wide range of pH and temperatures. Complete genome analysis of DRL-P1 confirmed the absence of any virulence factor such as toxins or antibiotic resistance genes. To the best of our knowledge, this is the first *Pbunavirus* to be isolated and characterized from India. In conclusion, our findings suggest DRL-P1 to be a potential candidate for therapeutic and sanitation applications.

## Materials and methods

### Phage isolation, purification, and preparation

Collection of wastewater sample was done from a community waste treatment facility (receiving human fecal matter), from Tezpur, Assam (26° 39′ 4.3848″ N and 92° 47′ 1.7268″ E). The host bacteria (*P. aeruginosa*) was isolated and grown on Cetrimide agar (Himedia, Mumbai, India). Briefly, the wastewater sample was spin down at 12,000×*g* for 10 min to remove debris and coarse matter. The supernatant was then serially passed through membrane filters of 0.45 μm and 0.22 μm pore size (Whatman filters, purchased from Sigma-Aldrich, St. Louis, USA). *P. aeruginosa* culture in the early exponential phase (approximately 10^7^ CFU mL^−1^) was infected with the filtrate obtained and allowed to infect the host cells at 37 °C overnight with shaking (180 rpm). The presence of lytic phages in the sample was identified through clearance of lawn in spot tests.

A single plaque was picked and suspended in TM buffer (50 mM Tris–HCl, 10 mM MgCl_2_, pH 7.4). The titer of the phage was determined by making serial dilutions of the released phages and using it to infecting fresh *P. aeruginosa* cultures in log-phase, followed by double-layer agar plating and incubation at 37 °C for 16 h. Plaques were visualized and photographed using a stereo-zoom microscope (Leica EZ4 HD, Leica, Wetzlar, Germany) and the diameter of plaques (*n* = 56) was measured through Leica Application Suite EZ version 2.1.0 (Leica, Wetzlar, Germany; https://www.leica-microsystems.com/products/microscope-software/p/leica-application-suite/). Subsequently, phage lysate containing 10^9^ PFU mL^−1^ was prepared by enrichment followed by PEG (polyethylene glycol) precipitation (8% wt/vol of PEG_8000_ and 1 M NaCl)^60^. Purified phage lysate was stored at 4 °C. A freshly prepared phage stock was sent for viewing under TEM.

This work was reviewed and approved by the DRL-IBSC (approval certificate DRL/IBSC/PROJ/10). All the microbiological manipulations were carried out inside a Class II A2 Biological Safety Cabinet (BSL2) (Esco, Singapore).

### Transmission Electron Microscopy (TEM)

Around 3 µL of purified bacteriophage suspension was gently placed on glow-discharged carbon-coated 300 mesh copper grids. After about 1 min, the remaining solution on the grids was wicked away with the help of a filter paper. The grid was then stained with 2% (wt/vol) uranyl acetate and air-dried. The negatively stained phage particles were visualized with an FEI Tecnai 12 BioTwin Transmission electron microscope (FEI, Netherlands) at an operating voltage of 100 kV. Virus particle dimensions were measured using the ImageJ computer program (version 1.53e, https://imagej.nih.gov/ij/)^61^, with the software scale set on the scale bar obtained from the electron micrographs.

### Antibiotic sensitivity assay and host range

Antibiotic sensitivity of *P. aeruginosa* strain used in this study was assessed using commercially available antibiotics coated Hexa discs G- minus 1 & G- minus 2 (HiMedia, Mumbai, India). Results were interpreted following the Clinical Laboratory Standard Institute (CLSI) guidelines as resistant, intermediate, or sensitive^62^. The following antibiotics were included in the assays: Ampicillin (AMP) 10 µg, Amoxyclav (AMC) 30 µg, Cefotaxime (CTX) 30 µg, Co-Trimoxazole (COT) 25 µg, Gentamicin (GEN) 10 µg, Tobramycin (TOB) 10 µg, Ceftazidime (CAZ) 30 µg, Ciprofloxacin (CIP) 5 µg, Amikacin (AK) 30 µg, Nitrofurantoin (NIT) 300 µg, Netillin (NET) 30 µg, Nalidixic acid (NA) 30 µg.

The host range of phage DRL-P1 was determined by spot assay and confirmed using the standard double-layer agar technique. Briefly, 5 µL of phage lysate (> 10^9^ PFU mL^−1^) was spotted over a lawn of bacteria mixed with top agar. After overnight incubation at 37 °C, plates were examined for plaques/ clear zones.

### Adsorption assay

For determination of the time required for the phage DRL-P1 to attach to its host, adsorption assay was performed according to Kim et al.^63^ with minor modifications. Briefly, exponentially growing host strain was infected with the phage at an MOI of 0.1 and was immediately aliquoted into separate vials. To see the effect of MgCl_2_ on phage adsorption, 10 mmol L^−1^ of MgCl_2_ was added into one aliquot, while an equal volume of sterile water was added to the other aliquot (no MgCl_2_). At 0, 5, 10, 15, and 20 min post-infection time intervals, 100 μL of samples were drawn from each experiment, immediately diluted in 900 μL of phosphate-buffered saline, and centrifuged at 12,000×*g* for 5 min. The titer of the non-adsorbed free phages in the supernatants so collected at different time intervals was determined by double-layer plaque assay.

### Single-step growth curve

A single-step growth curve was performed following Kim et al.^63^ to determine the phage latent period and burst size. In brief, 10 mL exponentially growing *P*. *aeruginosa* culture was infected with phage particles at an MOI of 0.1 and was allowed to adsorb for 15 min at 37 °C. Subsequently, cells were pelleted by centrifugation (12,000×*g* for 5 min) and unadsorbed phages were removed by washing the pellets with fresh TSB. Cell pellets were then resuspended in 10 mL fresh TSB broth and incubated at 37 °C. Cultures were incubated for 120 min and after every 10 min, a sample was taken for double layer plaque assay. Each experiment was conducted in triplicate.

### Temperature and pH stability assays

For thermal stability assays, equal volumes of TM buffer (900 µL) were aliquoted into 1.5 mL microcentrifuge tubes. All the tubes were kept at different temperatures (4 °C, 25 °C, 37 °C, 40 °C, 50 °C, 60 °C, and 70 °C) for 30 min. Subsequently, 100 µL of phage (~ 10^7^ PFU mL^−1^) was added into each tube, mixed gently, and incubated at their respective temperature conditions for 60 min. Similarly, for pH stability assay, 100 µL of phage (~ 10^7^ PFU mL^−1^) was added to microcentrifuge tubes containing 900 µL buffer solution adjusted at various pH (3.0, 4.0, 5.0, 6.0, 7.0, 8.0, 9.0, 10, 11, 12) and incubated for 18 h at room temperature. In both the studies, a double-layer plaque assay was performed and the percentage of surviving phages (as compared to phage stored in TM buffer pH 7.4 at 4 °C) was calculated by final PFU count over the initial PFU count^64^.

### Genome sequencing, annotation, and genome analysis

For extraction of nucleic acids, phage particles released from the lysis of the host cells were collected by gently rinsing the top layer of '*web pattern*’ plaque plates, using SM buffer. The high titer bacterial lysate was clarified by centrifugation at 14,000×*g* for 15 min at 4 °C and the clear supernatant was transferred to a fresh microcentrifuge tube. Subsequently, the supernatant was incubated with 50 U mL^−1^ of DNase I (Sigma-Aldrich, St. Louis, USA) and 40 U mL^−1^ of RNase A (ThermoFisher Scientific, Waltham, USA) for 2 h at 37 °C to remove contaminating host nucleic acids, and DNase I was then inactivated by incubation at 80 °C for 15 min. Capsid-protected phage DNA was released by proteinase K digestion for 2 h at 56 °C, purified by repeated cycles of extraction in Phenol/ Chloroform/ Isoamyl alcohol, precipitated using isopropanol, and finally dissolved in TE buffer^60^. The quantity and quality of the phage DNA preparation were evaluated spectro-photometrically (NanoPhotometer, Implen GmbH, Germany). The integrity of the DNA preparation was verified by electrophoresing an aliquot in 0.8% agarose gel, along with λ DNA/Hind III marker (Fermentas, Vilnius, Lithuania).

Purified phage DNA was sent to a commercial facility for NGS-based whole genome sequencing (AgriGenome Labs Pvt. Ltd., Kochi, India). Following standard quality evaluation, a paired-end library was prepared (Next Ultra, New England Biolabs, Massachusetts, USA) and library quality was evaluated on an automated electrophoresis platform (Tape Station, Agilent, Santa Clara, USA), followed by sequencing on Illumina HiSeq NGS platform (Illumina, San Diego, USA). After the sequencing run, adapter sequences were trimmed from the raw reads, reads with an average quality score of < 30 in any of the paired-end reads were filtered out as well as unique reads were removed. High-quality paired-end reads were then assembled de novo, using the Iterative Virus Assembler^65^. Analysis of features of the resulting phage genome including ORF prediction and annotation were accomplished using the GeneMarkS^66^ and PHASTER^67^ servers. Blastn (megablast) search was performed to find highly similar phage genome sequences in the NCBI GenBank^68^. BLASTX algorithm with E-value cutoff ≤ 10^–3^ was used to compare predicted genes with protein sequences submitted in the Uniprot database. A physical map of the annotated DRL-P1 phage genome was reconstructed using the SnapGene tool (trial version). The tRNAscan-SE program (http://lowelab.ucsc.edu/tRNAscan-SE/) was used to scan for potential tRNA genes in the genome^69^. Putative promoter regions were identified using the Neural Network Promoter Prediction program hosted on the Berkeley Drosophila Genome Project website (www.fruitfly.org/seqtools/promoter.html), with a minimum promoter score set at 0.9^70^. To identify Rho-independent transcription terminators, the ARNOLD server (http://rssf.i2bc.paris-saclay.fr/toolbox/arnold/index.php) was used^71^. The lifestyle of the phage was predicted using the PHACTS server (http://www.phantome.org/PHACTS/index.php)^72^. Antimicrobial resistance (AMR) genes and variants were predicted using the Resistance Gene Identifier (RGI) tool incorporated in the Comprehensive Antibiotic Resistance Database (CARD) server (https://card.mcmaster.ca/analyze/rgi)^73^.

For reconstruction of evolutionary history, complete genome sequences resulting from BLAST search and well-annotated reference sequences (RefSeq database^74^) were retrieved from the NCBI Virus database (https://www.ncbi.nlm.nih.gov/labs/virus/vssi/#/). Genomic or subgenomic sequences (DNA polymerase III/ terminase encoding genetic regions) were manipulated using BioEdit version 7.2.5 (https://bioedit.software.informer.com/download/)^75^. Multiple sequence alignments were done using the MAFFT online server (https://mafft.cbrc.jp/alignment/server/)^76^, allowing local adjustment of sequence orientation, during alignment. The Neighbor-Joining method was employed to infer evolutionary history. The Maximum Composite Likelihood (MCL) model was used to calculate evolutionary distances and bootstrap tests (1000 replicates) were performed to examine the reliability of clustering among the taxa. Evolutionary divergence (expressed in terms of base substitutions per site) between phylogenetically closely related sequences were estimated using the MCL model including 1st + 2nd + 3rd + Noncoding positions. Ambiguous positions were excluded during analyses. All the molecular evolutionary analyses were performed using the MEGAX program version 10.2.4 (https://www.megasoftware.net/)^77^.

Pairwise intergenomic similarities among the related phage genomes were calculated using the Virus Intergenomic Distance Calculator (VIRIDIC; http://rhea.icbm.uni-oldenburg.de/VIRIDIC/)^78^, which is based on the algorithm used by the ICTV (International Committee on Taxonomy of Viruses) to compute intergenomic similarities among Bacterial and Archaeal Viruses for taxonomic classification of phages. To elucidate the genetic relatedness of the phage DRL-P1 genome with other homologous phages, we used the progressiveMauve, version 2.4.0 (http://darlinglab.org/mauve/mauve.html)^79^ computer program, which employs progressive multiple genome alignment techniques to compare related genomes and effectively displays the relative location of locally colinear blocks (LCBs) among genomes, indicating genetic rearrangements, loss or gain, and segmental duplication of genetic regions.

To verify reticulate evolution, we reconstructed NeighborNet (NN) network tree using the SplitsTree4 computer program (version 4.14.6; https://uni-tuebingen.de/en/fakultaeten/mathematisch-naturwissenschaftliche-fakultaet/fachbereiche/informatik/lehrstuehle/algorithms-in-bioinformatics/software/splitstree/)^37^. Genetic differences were estimated using the Kimura-2 parameter algorithm implemented in the program. Parsimony uninformative sites and gaps were excluded from the analysis. To examine the probability of recombinations in the DRL-P1 genome, aligned complete genome datasets were analyzed through the Recombination Detection Program, an RDP4 computer program (version 4.95; http://web.cbio.uct.ac.za/~darren/rdp.html) set at default values and standard Bonferroni correction^80^. The RDP4 program incorporates RDP, GENECONV, BOOTSCAN, MAXCHI, CHIMAERA, SISCAN, 3SEQ, and PHYLPRO algorithms for analysis of recombination and breakpoints. Recombination events predicted by the program were verified by examination of the breakpoint plots and UPGMA phylogenetic trees generated by the program. Events predicted by at least three different algorithms and had adequate statistical support (*P *< 0.01) were considered as true events.

### Sequence accession number

The genome sequence of DRL-P1 is available in the NCBI GenBank under accession number MN564818.

### Fomites decontamination assay

For a demonstration of decontamination of fomites by the phage DRL-P1, in vitro assays were performed using contaminated glass coverslip as model fomites, following Jensen et al.^43^. Briefly, fresh *P. aeruginosa* culture was diluted to 10^6^ CFU mL^−1^ and 10 µL of diluted culture was spread over the coverslip and dried for 30 min at room temperature inside a biosafety cabinet. Thereafter, 100 µL of phage lysate was added at MOI:1.0, 0.1, 0.01 & 0.001 and phage action was allowed for 45 min. Subsequently, the coverslip was placed in 500 µL of fresh TSB, vigorously vortexed for 10 s to dislodge the attached bacteria from the surfaces. A control treatment (MOI:0) was performed using sterile phage buffer instead of phage lysate. Cultures were serially diluted and plated on TSA agar and incubated overnight at 37 °C. Percent decontamination was calculated for each MOI by calculating percent reduction in the bacterial count in comparison to control.

### Phage lytic activity in vitro

Phage kinetics was performed according to a previous study^81^ with minor modifications to study the in vitro lysis of bacteria through a change in absorbance of optical density. Briefly, 180 µL of bacterial culture in the log phase (10^5^ CFU mL^−1^) was aliquoted into wells of a 96-well microtiter plate. 20 µL of bacteriophage suspensions were added to wells at different MOI (100, 10, 1, 0.1, 0.01, 0.001). Cetrimide broth without *P. aeruginosa* was used as a negative control, *P. aeruginosa* bacteria without phage was a positive control for the experiment. The microtiter plate was incubated at 37 °C inside a programmable real-time multi-mode microplate reader (Varioskan LUX, Thermo Scientific, USA) and the change in absorbance at OD600 was automatically recorded at 10 min intervals for 390 min. Instrument set-up, run, data acquisition, analyses, and graphical presentations were done using the accompanying software, SkanIt Research Edition version 5.0 (Thermo Scientific, USA; https://www.thermofisher.com/order/catalog/product/5187139#/5187139). Three independent trials were conducted to replicate the experiment.

### Biofilm degradation assay

The biofilm degradation potential of DRL-P1 was determined using a previously published protocol with slight modification^81^. Briefly, aliquots (200 µL) of *P. aeruginosa* culture (1.8 × 10^6^ CFU mL^−1^) were incubated in 96-well polystyrene microtitre plates for 24 h at 37 °C. After incubation, planktonic cells were removed carefully and 200 µL aliquots of DRL-P1 diluted in cetrimide broth were added to each well at different MOIs (10, 1, 0.1, 0.01, and 0.001). Inoculated bacteria without phage were taken as a positive control whereas, cetrimide broth without bacteria was used as a negative control. The wells were stained at 12, 24, and 48 h of incubation. For staining biofilm, 1% w/v crystal violet (LOBA Chemie, India) was added and allowed to stain for 20 min at room temperature. Subsequently, the wells were washed thrice with PBS to remove the unbound dye, followed by the addition of 1% SDS (w/v) to dissolve the bound dye. The absorbance was determined at OD595 and data analyzed using microplate reader and accompanying software (Varioskan LUX & SkanIt). The percentage of biofilm clearance was calculated according to a previous paper^82^.

### Lyophilization of phage lysate and encapsulation on alginate

Lyophilization and phage encapsulation on alginate was performed according to Gonzalez-Menendez et al.^83^ with slight modifications. Phage lysate (~ 10^9^ PFU mL^−1^) was diluted (1:1 v/v) in 22% skimmed milk and 1.6 M sucrose and the diluted phage were allowed to freeze in 2 mL vials for 24 h at -80 °C. Bacterial cells in log phage were resuspended in skimmed milk lysate to make a final dilution of 11% (v/v) skimmed milk and 0.8 M sucrose. Samples were lyophilized in a laboratory freeze dryer (Labconco, Kansas City, USA). The lyophilized preparation was reconstituted with 2 mL sterile TM buffer and phage titer were calculated using the standard method.

Encapsulation of phage DRL-P1 within alginate beads was done following a previously described method, with minor modifications^55^. In brief, a phage-alginate mixture was prepared in 50 mM TM buffer (pH 7.5) containing ~ 10^8^ PFU mL^−1^ of DRL-P1 and 2% (w/v) sodium alginate (Himedia, Mumbai, India). The mixture was stirred continuously for 1 h at 500 rpm at room temperature and subsequently added dropwise into 0.1 M CaCl_2_ solution to prepare phage-loaded alginate beads. The beads were incubated for 30 min at room temperature with gentle shaking to complete the cross-linking of calcium alginate bead walls. After another 30 min of incubation at room temperature, beads were gently washed with nuclease-free water and finally stored at 4 °C, until use.

### Statistical analysis

The unpaired t-test was used for statistical analysis in this study. The level of significance was set at (*P* ≤ 0.05). GraphPad PRISM (Trial version 7.05; https://www.graphpad.com/scientific-software/prism/) (GraphPad Software, La Jolla, USA) for windows was used to analyze the data.

## Supplementary Information

Supplementary Information.
