# Supplementary material for: Isolation and characterization of a lytic bacteriophage against Pseudomonas aeruginosa
Source: Sci Rep. 2021 Sep 29;11:19393. doi: 10.1038/s41598-021-98457-z (PMC8481504; doi:10.1038/s41598-021-98457-z)
Supplement: Supplementary file 1 — Supplementary Information. [file 41598_2021_98457_MOESM1_ESM.pdf]

Isolation and characterization of a lytic bacteriophage against *Pseudomonas aeruginosa*

Sonika Sharma[1¶*], Sibnarayan Datta[1¶*], Soumya Chatterjee[1], Moumita Dutta[2], Jhuma Samanta[1], Mohan G Vairale[1], Rajeev Gupta[1], Vijay Veer[1], Sanjai K Dwivedi[1]

[¶]Sonika Sharma and Sibnarayan Datta contributed equally to this work.

[1]Defence Research Laboratory (DRL-DRDO), Tezpur, Assam.
[2]National Institute of Cholera and Enteric Diseases (ICMR-NICED), Kolkata, West Bengal.

*Corresponding authors:
ssharma@drl.drdo.in (SS)
sndatta@drl.drdo.in (SD)

Table S1. Representing P. aeruginosa sensitivity and resistance against different antibiotics and results are interpreted according to Clinical and Laboratory Standards Institute (CLSI) guidelines R: Resistance I; Intermediate S: Sensitivity.

Antibiotic Concentration	R	I	S
Ceftazidime (CAZ) 30µg	R		
Ciprofloxacin (CIP) 5µg			S
Amikacin (AK) 30µg			S
Nitrofurantoin (NIT) 300µg	R		
Netillin (NET) 30µg		I	
Nalidixic acid (NA) 30µg	R		
Ampicillin (AMP) 10µg	R		
Amoxyclav (AMC) 30µg			S
Cefotaxime (CTX) 30µg			S
Co-Trimoxazole (COT) 25µg	R		
Gentamicin (GEN) 10µg			S
Tobramycin (TOB) 10µg		I	

Table S2. Table representing host range of DRL-P1 bacteriophage (+) show zone of lysis and (-) show absence. Pseudomonas isolates IS (1-9) was isolated from the soil samples of Arunachal Pradesh (AP), India. MTCC-Microbial Type Culture Collection and Gene Bank (Institute of Microbial Technology, Chandigarh).

**S. No.**	**Isolate name**	**Place of Origin/ Source**	**Infection**
1.	*Pseudomonas aeruginosa* IS1	Soil, AP	+
2.	*Pseudomonas aeruginosa* IS2	Soil, AP	+
3.	*Pseudomonas aeruginosa* IS3	Soil, AP	+
4.	*Pseudomonas aeruginosa* IS4	Soil, AP	+
5.	*Pseudomonas aeruginosa* IS5	Soil, AP	+
6.	*Pseudomonas aeruginosa* IS6	Soil, AP	+
7.	*Pseudomonas aeruginosa* IS7	Soil, AP	+
8.	*Pseudomonas aeruginosa* IS8	Soil, AP	+
9.	*Pseudomonas aeruginosa* IS9	Soil, AP	+
10.	*Pseudomonas aeruginosa*	MTCC (No. 1688)	+
11.	*Escherichia coli*	MTCC (No. 443)	-
12.	*Vibrio cholera (Classical 01)*	MTCC (No. 3904)	-
13.	*Bacillus megaterium*	MTCC (No. 428)	-
14.	*Shigella flexneri*	MTCC (No. 1457)	-
15.	*Bacillus subtilis*	MTCC (No. 1305)	-
16.	*Salmonella enterica* Typhimurium	MTCC (No. 1251 & 1252)	-
17.	*Streptococcus pyogene*	MTCC (No. 442)	-
18.	*Klebsiella pneumoniae*	MTCC (No. 8911)	-

Table S3

Table S3. Promoters and terminators predicted in the DRL-P1 genome.

Promoter predictions for DRL-P1 (Forward strand):

Start	***End***	***Score***	***Promoter Sequence***
516	561	0.98	GCTCGGTTTTGAATTCGCAATGCAGCCTTCATAATCCGGACCCGAACATC
1733	1778	0.97	TCTTTGATGATATCATTGATGTACGATGGGACATTTATCGGCTCGGCCTT
3925	3970	0.97	CGACTTGGAAGTGATGGCGAAAGTCGAGGTATTGTCATTGAAAGCATTCA
4576	4621	0.95	ACATCAATTGAAGGGCAGAATACCTCAACGATGATCTCCATAGGGTGGAC
5045	5090	1.00	CTTTTTAGAATTCAGAAGGATGCGAAGATTAATATCATCTTCACTCCCTG
9689	9734	0.99	CTCTAGTTGAAAAACCCTTCCGGTCCAGATAAACTGGTCACCGGCCAAAT
9785	9830	0.97	ATTTCGTTGAAACTCCAGATTGAACTGGACATACTGATCGCGACGAACCC
14126	14171	0.96	ACCGAGTTGACCGGCAGAATATTGGAAAATACCATAAGAGCTGATTGGAC
14320	14365	0.95	CGACTGTTAACTTACACCATAATTTGTATTATGATCTAATTCCCAGATTT
14712	14757	0.99	ATTTTTAGTCAGAATCCGAATTTTCGCGGCATCATTTGCCGAAAACAATC
15779	15824	0.96	GCCTTTTCAATTTCACTTCGATAATCGGCGATCATGAGTTTGGTAATATC
18358	18403	0.97	TAATTCTTTACGGGAACTTTTCGGCAGATTAGACTCTAATTATCAACCAA
21541	21586	0.96	GGATTTGCTTCCATCACTCTTCCATCCACGATCATCGATTCGATTTCGAG
24331	24376	0.96	GACCTATTTGGCCGATAACGGCTATAACGTAACATTCAGCAAAGGTGAAA
26592	26637	0.96	CCACTTTGAGAAATATTTCACAATTCTGCTTTACTTCCATTCTGAATAGC
28986	29031	0.96	ATCTTGGAAGTCATTGGCGTAGAAGAAGTCAAAATCGACGGCGTGACTCG
29500	29545	0.98	ATTCTTTGAAGTCGAACCCTACATAAAGGAACAATCATGAAACTCTACCA
30862	30907	0.97	ATGTTGGCTTGTGAACTGACCGTAGGCGATGAAATACTCGCCCATGTGGA
32404	32449	0.98	TAAATGTAGACACGCAGCATCGATCTAGGCATAATCTCTTTCAACGGGGC
34389	34434	1.00	TAATTTTTAAAGATAGGTAACGTCCGTGATAGATTTTTTCTATTAGGATT
36871	36916	0.98	CGTTTTGCATATTGTCTACTCCTCACCGATATGACGGCGGTTGATTCTTT
37162	37207	0.96	CATTTTGTATTTCGTGCAGATGTGCTCGGCATACTTCAGCCATACTTGGT
37393	37438	0.98	CTTGTTGTTGAAGCCGAATGGCATGCGACCATAACGGCTGACGTCTTTGA
40181	40226	0.99	GTTTTGGAGGGCAGTACAGGCGGCGGCGCTAGTATCGGCCACTTTCCTGC
40725	40770	0.96	GCGTTGGAGAACGCAAATATGTCAGCATCTAACTTCACCGTCGATCAGAT
41833	41878	0.99	TGTTGGAAGAAAACTTACCGTCCATTCCTTATAGTCAATTGATGTATACT
50661	50706	0.97	AGGATTGGGAGAAAGTTGCCGCGAGTTTGTCAAATTCTGAGGTTCAATTC
52469	52514	0.97	ATATATTTCGAAATGTCGCCGGAGCAAAGGAAGATTTATCAGCGCGTTGA
52700	52745	0.99	GGTCTGTTGGAGGAAGACCCGGAACGGTCTATCATCATCTGGGCCATGCG
53266	53311	1.00	AAGAATTGGAAGTTCCGGAGTATCTGAAGGACAATGCGCCGGGCGAAATT
53601	53646	0.99	AAGTGGCTGGAAGACACAGACAACGACGGCATCATCAAGACCAAAGTTCT
53882	53927	1.00	AATTTGCTTTACTTCGCTGCAACTTCCGTTATTATAAACCCACAGTTAGC
58790	58835	0.96	TTGTAGTTTGCGGAGCAAGACGAGTAGATGAGAATGGAACCGTCCACAAC
59566	59611	0.98	GGCTTTGATGGTCAGCTTGGTGTTCGGGTTAAAGTTCCACGGCCCCCAGA
63664	63709	0.96	GGTATTGTTTTCGATAACGTTGACCGAGGTAACATTGGGTACGGCGCTGA

64617	64662	0.98	CGATTGGATGACCATCGCGGCCGAGTCTTCAGAATTGATGGTGTATCGGC
64914	64959	0.98	TTGGATTGTGGCGATATTTTCGGCCCGATCATATTGAACAACCTGGGCGG

Promoter predictions for DRL-P1 (Reverse strand):

Start	***End***	***Score***	***Promoter Sequence***
66239	66194	0.96	CAGAGTATTTGTCGGCGAAGTGGCCATTGTTGATATCATTTCGCCGCCA
66220	66175	0.99	AGTGGCCATTGTTGATATCATTTCGCCGCCACCGGATATTGGAATTCGCA
66073	66028	0.96	TGAAATGGGGTTGAACTTCATCTGCGATACCAGCTACAATGATCAAGTTT
65722	65677	0.98	AGAGTATGATTTGGCCAGTCGGGATCGGCCTTTCTATATCAAAGTCATGG
65367	65322	0.98	TCCTCAAAGATTGGACAGGGGTCAAAATTCCAGCCAGATTTGTCGAAGAT
64703	64658	0.98	TCCACAAATTTGAACATGGAATGTTCATCCCGGATGTATTCCGCCGATAC
64383	64338	0.96	TTTTGATTTTTCTGATTTAACAGCGGGGGGGGGTTGTAATGGCTAATTAT
63281	63236	0.97	GTCTGCATTTGAAGTGGCTGGGGCTATCGCTCGCGAGATTCCCGGTATCT
62947	62902	0.98	TTTTCAATTTGAAGACTGCCAACCCGTTCGGCCTTATGGTGTGGTGCATC
62167	62122	0.99	GCGATCAGATGTTGGACAGCAGCGTGGTAAGCGCCAGAATGATTCGTTTG
61004	60959	0.95	TGGCATGTTGGAGGCGAAATCTTGGATATCCGGGTCCAATACGTTCTGCG
59214	59169	0.96	AAGTGGATTTGAACGCCGCGAACGCCACCAACGGCAACATGATCTTCAAC
58193	58148	0.96	TCAAGTGGCTGGATGACGTCCAGGCGGCCATGGATAAGTTCCACATCGAG
50656	50611	0.98	TGAAGCGTTTTAAGATGGGAGCCGGCCAGCTCTTTATATTGCGCCTTCAA
49305	49260	0.99	TGATCGAGTTTTTGATGTTGTCGGTATCATGGAATGGAATGCCGAAGCGC
46645	46600	0.99	GGAAATAGTTGTTGTCGGTTCGGCCGGTAATGAAGAAAATCATCAGGTCG
42061	42016	0.97	GGGATGGCTTATTGGCCTGTGAGGGGATTATCTCTAAACTAACTGAAGAA
41802	41757	0.99	CTGCGACTTGTGGAACGTCATCGCCCAGCCGAAGTCGAATCCGCCAATGG
36246	36201	0.97	CAGCTCGACTTCTGACAGCAACTATCTCGCGGTGTACGATACTTTGTTCT
35570	35525	0.96	GACCAGAAGTTTGAGCCGGGCGAGACGATCAAGCGACAATGGATTACCAT
34640	34595	1.00	GTGTATGATTGTTGGATTTTGCGTGAAATGTTGAGAAATTGCGGGTTTGA
34350	34305	0.98	TTTAGTCTTTGAAAGCCTCGCGGCGCTAAGCTTGTACATTACCCGGCAAG
29137	29092	0.96	TCGATCATCTGGAAATCGAACATTGCATAGCTCATACCATCACCATGTCG
27229	27184	0.96	CGTCGATGCTGTTGAAAGGGATTAGCTTGGACACGACAATTCTCCTGTGA
26679	26634	0.98	CTCTGTTTTGGAGTGTTTCGCGTTTCGATGAAGAGATTATGACGCTATTC
26637	26592	0.98	CGCTATTCAGAATGGAAGTAAAGCAGAATTGTGAAATATTTCTCAAAGTG
26306	26261	0.98	CGTTGGTCAGAATGGAAGTAAAGTGTATTAACAATAAAATTATGTTCACC
22867	22822	0.98	CGGTGATTGTTGATGCCACCGACGAAATCCATGATGCAATCATTATACGT
20379	20334	0.99	CTCCTGTTTGGAAAGTTCGTTTCGATGGGTTGACTATACTCCATAAATGG
18308	18263	0.95	GTTTGAATCTCTTTTGAACGTTTGATGTTTCCCCTATAATAAGCGCACAC
18251	18206	0.97	AACCGCATGGAATTAAAATGTTTAAACTTTCCTGGATATTCGGGCGCAAA
17518	17473	0.99	TGAGCCCGATTTTTGGATCATCAGCGGGAAGAAGTATCATCGCAGCCATC
14765	14720	0.96	CTCAATCCTGATTGTTTTCGGCAAATGATGCCGCGAAAATTCGGATTCTG
14668	14623	0.97	TAGCGGTAATTTGGAAAGCCTACTGCCGCAAGGCTTTAACAGGGGAAATT

14487	14442	0.99	TTCAATTTTTAATTGGTAAATTGGTAATTTGAATTAGTTTAAAGGTTGAA
11890	11845	0.95	TGCCTGGGTTCGTGAAGGTCATGACCGCCGCGCGGAAAATCGATGAGATC
9394	9349	0.97	CCGGCAACCGATTGGTATCTGGACAACGCAGCCAGAAAATATGTAGAAAT
9329	9284	0.99	CGAGACGACTTTTCAGATAAGTTCCCTTCATTGGCAAAATCCTGATCTGG
8805	8760	0.98	GGGTCGGTTTGTTGGCGTTCGGTTCGTCAGAAACGATTTTCCGGGAGAAA
8697	8652	0.99	TTGGAACTTGTTTTCAGCAGATCGCGCCGCGATCCAAAATCGAATAGCCA
7006	6961	0.97	GGCTTGACCGAGTGGAAGGCCAACTACACCCTGATCTATTCGAAGGGCGA
3736	3691	0.99	AATTTGCTAGACTTGGGAGCCACCGGCGATTCGATATCTTCGGCCGCAAA
3212	3167	0.96	CATGTCTAGTGATTTGGATGAATTCATACTTCGGTATGAGGCCGACACGG
349	304	0.96	CCAGAGTATTTGTCGGCGAAGTGGCCATTGTTGATATCATTTCGCCGCCA
330	285	0.99	AGTGGCCATTGTTGATATCATTTCGCCGCCACCGGATATTGGAATTCGCA
183	138	0.96	TGAAATGGGGTTGAACTTCATCTGCGATACCAGCTACAATGATCAAGTTT

Table S4: Rho-independent terminators predicted in the DRL-P1 genome.

Rho-independent terminators

Position		*Strand*	*Sequence*	
3219	Both	+	ATTACTCCGGCTTGCTGGCGAGTCCTTCGGCGATTTTCATAGCTG	-6.10
4677	Both	-	TTGAGGATAAGCCGGGTTAACACCCGGTTTTCTTCTGTA	-10.10
5225	Rnamotif	-	GATAGTTTCAACCTCGTCGTTCCCGATGGGGTTTTCCTTGTCG	-11.00
10356	Both	-	AGTCCTATCCGGCTGTGCGCCTGCAGCTTTATTTCGACA	-5.40
10957	Both	-	TGACTCACCAAAGGCCGGGCTTCCGGCCTTTGTTCACTCTGA	NA
20535	Rnamotif	-	GGTACAGGCCGGTGCCTGCGCATTTGGGGCACTTTTCGCGATAC	-8.80
20874	Rnamotif	-	CGCGGCCACTTTCTCGCGATGTTTGGTGAGATTTTCGATGGCT	-8.00
22336	Both	-	GTGCGCCAGGCTTCAGCTTGGGGTGGCTGAGTTTTGGCTCTAC	-8.50
24764	Both	-	AACACAGAAAAGCCCGGAGCGATCCGGGCTTCTGGTCTTACT	NA
24766	Both	+	TAAGACCAGAAGCCCGGATCGCTCCGGGCTTTTCTGTGTTT	-13.80
30968	Both	-	ACCGCCGACGAGTGCCGAGACTTCGGCGCTTACTTTGTTGT	-11.30
32320	Both	-	GGATGTGAAAACACCGCAGGACAGGCTGCGGTGTTTTCTGGACGAT	-15.50
32322	Both	+	CGTCCAGAAAACACCGCAGCCTGTCCTGCGGTGTTTTCACATCCG	-14.00
33120	Erpin	-	GAAACGATAAAGCCCCGGACTCTAGTTCAGAATCCGGGGCTTTCTTTTGGGT	-15.70
33122	Both	+	CCAAAAAGAAAGCCCCGGATTCTGAACTAGAGTCCGGGGCTTTATCGTTTCTG	-15.70
34761	Rnamotif	-	GGAGTGAATTAGGCCGCCTTCGGGCGGTCTTTTCTTTGGTG	-13.70
43011	Both	+	TAGCTCTTCAAGGGACTCATGAAAATGGGTCCCTTTTTTATCCTC	-12.60
43392	Rnamotif	-	GTAGTCGGCCGGCGCGATCCAGCGCTTTTCGGTCGCC	-7.10
44492	Rnamotif	-	GTCGCCTTCTGGCCCGACGATCCGGGCTGTTATTGTGAG	-9.50
48764	Both	-	TAGGTGGATCATTGGCGCTTTTGCCAGTTTTACATCGAC	-5.50
53851	Both	-	CAGCGAAGTAAAGCAAATTACATGTTAAGATTTGCTcTTTTTCACCTTCG	-6.10
54865	Rnamotif	+	CAACGGCCAGAGAAAGGCGCTGTAATGGCGCCTTTCTTTTGAGGAATC	-11.10
56082	Both	+	ACCGCCATTCAGCCCGGTCCGCCGGGCTTCTTTCTGGAG	-12.20
57052	Erpin	-	AAAACGAGAAAACCCGGATCGCCTTTAGGATAAGGCGTCCGGGTTTATTTCGATCTA	-11.50
57054	Erpin	+	GATCGAAATAAACCCGGACGCCTTATCCTAAAGGCGATCCGGGTTTTCTCGTTTTAA	NA
57545	Both	-	CGAAAAGAAAAGGCCGCTTATTCAGCGGCCTTTTTGCTTTCC	-13.30
57546	Both	+	GAAAGCAAAAAGGCCGCTGAATAAGCGGCCTTTTCTTTTCGG	-14.70

Total number of predicted transcription terminators: 27

Table S5. Genus and species cluster of DRL-P1 sequences as determined in comparison with 100 top BLASTn hits including 37 RefSeq and 63 yet unclassified sequences.

genome	species_cluster	genus_cluster
KF981875_SPM1	1	1
NC_007810_F8	1	1
MT119364_elmo	2	1
MT119370_steven	2	1
KR054028_DL52	2	1
MN564818_DRLP1	2	1
MW557846_zikora	2	1
KT372690_Gallinipper	3	1
KT372694_Nemo	3	1
KT254133	3	1
KT372697_Smee	3	1
KT372691_Jollyroger	3	1
KT254131	3	1
KT254132	3	1
KT372698_Triton	3	1
KT372695_Nessie	3	1
KT372693_Kula	3	1
KT372696_Poseidon	3	1
KT372692_Kraken	3	1
KT254130	3	1
NC_011810_PB1	3	1
MH536736_E79	3	1
MT119367_misfit	3	1
MT133563_billy	3	1
KU198331_NP3	4	1
NC_011756_SN	4	1
KX171208_vB_Pae436M8	5	1
NC_041865_phiKTN6	5	1
MT133562_willy	5	1
NC_041870_vB_PaeM_CEB_DP1	5	1
MT119368_shane	5	1
NC_011166_LMA2	5	1
LC102730_S121	6	1
LC472883_S123	6	1
MT118299_Epa25	6	1
MW595221_vB_PaeM_V524	6	1
MT118291_Epa12	6	1
MT118298_Epa21	6	1
NC_019935_KPP12	7	1
LC105987_KPP22	7	1
LC105988_KPP22M1	7	1
MT413450_Epa15	8	1
NC_042079_vB_PaeM_E217	8	1

MT119376_chunk	8	1
LC472884_S50	8	1
MT118297_Epa20	8	1
NC_048699_vB_PaeM_LS1	8	1
MT119363_debbie	8	1
MT349888_PHW2	8	1
MT119365_goodold	9	1
LN610588_vB_PaeM_PAO1_Ab29	9	1
NC_026586_vB_PaeM_PAO1_Ab27	9	1
LT594786_PII10A	10	1
MK318076_vB_PaeM_fHoPae01	10	1
MK837010_Pa204	11	1
NC_050145_PaGU11	11	1
MN131141_PaP1_EPu2019	12	1
MN131143_PA11P1	12	1
NC_028971_DL68	13	1
MN615702_vB_PaeM_SMS29	13	1
MN615700_vB_PaeM_SMS12	13	1
MN615701_vB_PaeM_SMS21	13	1
MT108726_Epa6	14	1
NC_048744_EPa61	14	1
MT118303_Epa39	14	1
MT119375_chumba	14	1
MT108729_Epa22	15	1
MT933737_PASA16	15	1
NC_050149_vB_PaeM_USP_1	15	1
NC_042080_vB_PaeM_E215	16	1
MT118290_Epa10	16	1
MT119369_sortsol	16	1
MT119366_jett	17	1
MT119372_cory	18	1
MT133561_goonie	19	1
MT491205_vB_PaeM_USP_2	20	1
MT491206_vB_PaeM_USP_3	20	1
MW406974_TH15	21	1
MW595220_vB_PaeM_V523	22	1
NC_011165_LBL3	23	1
NC_011703_141	24	1
NC_017674_JG024	24	1
NC_019451_NH4	25	1
NC_026600_vB_PaeM_C114_Ab28	26	1
NC_028745_DL60	27	1
NC_028939_vB_Pae_PS44	28	1
NC_041902_PA5	29	1
NC_048626_PA01	30	1
NC_048662_R12	31	1
NC_048663_R26	32	1

NC_048675_BrSP1	33	1
NC_050147_Epa13	33	1
NC_048676_SL1	34	1
NC_048745_vB_PaeM_SCUTS1	35	1
NC_048806_PA8P1	36	1
NC_050143_datas	37	1
NC_050144_Epa14	38	1
NC_050146_Epa7	39	1
NC_050148_Pa193	40	1
NC_050150_antinowhere	41	1
NC_050151_crassa	42	1

Table S6. Intergenomic similarity among 100 top BLASTn hits including unclassified sequences.

**genome**	**MN564818_DRLP1**
**MN564818_DRLP1**	**100**
**MW557846_zikora**	**97.493**
**KR054028_DL52**	**96.782**
MT119367_misfit	96.487
**MT119364_elmo**	**96.432**
**MT119370_steven**	**96.033**
MT133563_billy	95.964
MT119375_chumba	95.865
NC_011810_PB1	95.339
KT372692_Kraken	95.337
KT254130	95.337
KT372695_Nessie	95.333
KT372693_Kula	95.333
KT372696_Poseidon	95.333
KT254131	95.324
KT372698_Triton	95.324
KT254132	95.32
KT372691_Jollyroger	95.318
KT372697_Smee	95.303
KT254133	95.28
KT372694_Nemo	95.261
KT372690_Gallinipper	95.247
MT119372_cory	95.125
MH536736_E79	94.928
MT118303_Epa39	94.919
NC_048663_R26	94.11
NC_007810_F8	94.069
NC_048744_EPa61	93.948
MT108726_Epa6	93.943
KF981875_SPM1	93.906
NC_026600_vB_PaeM_C114_Ab28	93.855
NC_028745_DL60	93.284
NC_050150_antinowhere	92.998
NC_050143_datas	92.796
NC_050144_Epa14	92.742
NC_050151_crassa	91.523
NC_048626_PA01	89.819
NC_048745_vB_PaeM_SCUTS1	89.169
NC_011165_LBL3	88.915
NC_041902_PA5	88.513
NC_028939_vB_Pae_PS44	87.914
NC_050146_Epa7	87.392
NC_048662_R12	87.278
NC_041865_phiKTN6	87.138
LC472883_S123	87.12
LC102730_S121	87.118
MW406974_TH15	87.096
NC_042079_vB_PaeM_E217	87.004
NC_026586_vB_PaeM_PAO1_Ab27	86.779
LN610588_vB_PaeM_PAO1_Ab29	86.763
MT119365_goodold	86.758
MT413450_Epa15	86.706

NC_041870_vB_PaeM_CEB_DP1	86.698
KU198331_NP3	86.679
MT119366_jett	86.653
NC_011703_141	86.643
LC105987_KPP22	86.523
LC105988_KPP22M1	86.521
NC_050145_PaGU11	86.503
KX171208_vB_Pae436M8	86.483
MT118298_Epa21	86.391
NC_048675_BrSP1	86.342
MT349888_PHW2	86.312
MT133562_willy	86.299
NC_042080_vB_PaeM_E215	86.238
MT108729_Epa22	86.143
MW595221_vB_PaeM_V524	86.142
MK318076_vB_PaeM_fHoPae01	86.135
LT594786_PII10A	86.116
NC_048699_vB_PaeM_LS1	86.071
MT119368_shane	86.039
NC_048676_SL1	86.032
MT118291_Epa12	86.004
MT118297_Epa20	86.001
NC_019451_NH4	85.99
MN131141_PaP1_EPu2019	85.979
MT119363_debbie	85.953
NC_019935_KPP12	85.917
NC_050148_Pa193	85.843
MT491205_vB_PaeM_USP_2	85.84
LC472884_S50	85.806
MT119369_sortsol	85.796
MT491206_vB_PaeM_USP_3	85.769
MT118290_Epa10	85.736
NC_011166_LMA2	85.715
MT119376_chunk	85.652
MW595220_vB_PaeM_V523	85.618
MT118299_Epa25	85.61
NC_028971_DL68	85.605
NC_050149_vB_PaeM_USP_1	85.555
NC_050147_Epa13	85.528
MK837010_Pa204	85.452
MT133561_goonie	85.414
NC_011756_SN	85.371
MT933737_PASA16	85.044
NC_048806_PA8P1	85.038
MN131143_PA11P1	84.92
MN615701_vB_PaeM_SMS21	84.738
MN615700_vB_PaeM_SMS12	84.737
MN615702_vB_PaeM_SMS29	84.723
NC_017674_JG024	84.707
